# AXL Overexpression in Tumor-Derived Endothelial Cells Promotes Vessel Metastasis in Patients With Hepatocellular Carcinoma

**DOI:** 10.3389/fonc.2021.650963

**Published:** 2021-05-27

**Authors:** Zong-Tao Chai, Xiu-Ping Zhang, Jian-Yang Ao, Xiao-Dong Zhu, Meng-Chao Wu, Wan Yee Lau, Hui-Chuan Sun, Shu-Qun Cheng

**Affiliations:** ^1^ Department of Hepatic Surgery VI, Eastern Hepatobiliary Surgery Hospital, Second Military Medical University, Shanghai, China; ^2^ Department of Biliary Surgery I, Eastern Hepatobiliary Surgery Hospital, Second Military Medical University, Shanghai, China; ^3^ Department of Liver Surgery and Transplantation, Liver Cancer Institute and Zhongshan Hospital, Key Laboratory of Carcinogenesis and Cancer Invasion, Fudan University, Ministry of Education, Shanghai, China; ^4^ Faculty of Medicine, The Chinese University of Hong Kong, Shatin, China

**Keywords:** AXL, endothelial cells, vessel metastasis, portal vein tumor thrombus, hepatocellular carcinoma

## Abstract

Portal vein tumor thrombus (PVTT) is one of the most serious forms of hepatocellular carcinoma (HCC) vessel metastasis and has a poor survival rate. However, the molecular mechanism of PVTT has not yet been elucidated. In this study, the molecular mechanism of AXL expressed in tumor-derived endothelial cells (TECs) in vessel metastasis was investigated. High AXL expression was observed in TECs, but not in the tumor cells of HCC patients with PVTT and this was associated with poor overall survival (OS) and disease-free survival (DFS). AXL overexpression was positively associated with CD 31 expression both *in vitro* and *in vivo*. AXL promoted the cell proliferation, tube formation, and migration of both TECs and normal endothelial cells (NECs). High expression of AXL in TECs promoted the cell migration, but not the proliferation of HCC cells. Further studies demonstrated that AXL promoted cell migration and tube formation through activation of the PI3K/AKT/SOX2/DKK-1 axis. AXL overexpression in HUVECs promoted tumor growth and liver or vessel metastasis of HCC in xenograft nude mice, which could be counteracted by treatment with R428, an AXL inhibitor. R428 reduced tumor growth and CD 31 expression in HCC in PDX xenograft nude mice. Therefore, AXL over-expression in TECs promotes vessel metastasis of HCC, which indicates that AXL in TECs could be a potential therapeutic target in HCC patients with PVTT.

## Introduction

Hepatocellular carcinoma (HCC), the most frequent type of pathological liver cancer, often extends into the portal vein branches. The incidence of portal vein tumor thrombus (PVTT) has been reported to be 44% to 62.2% in patients with HCC (1–3). The current treatment for HCC patients with PVTT remains controversial. Studies from China and Japan have demonstrated that hepatectomy is associated with better survival outcomes than non-surgical treatment in selected HCC patients with PVTT limited to a first-order branch of the MVP or above (1, 4–7). Nonetheless, the Barcelona Clinic for Liver Cancer Staging System and Treatment Guidelines consider that HCC patients with PVTT have a low chance of a cure (8), and sorafenib is the only recommended therapeutic option (9, 10). Therefore, PVTT is an extremely poor prognostic factor for HCC patients (2, 3, 11, 12). The median survival time of HCC patients with PVTT ranges from 2.7 to 4.0 months, if left untreated (2, 13). However, the underlying molecular mechanisms of PVTT of HCC remain largely unknown.

PVTT is the most serious type of vessel metastasis in HCC patients. Receptor tyrosine kinase AXL belongs to the Tyro-Axl-Mer (TAM) receptor tyrosine kinase family (14–17). AXL is mainly activated through auto-phosphorylation of its intracellular tyrosine kinase domain, thus inducing PI3K/AKT and Wnt signaling in mesothelioma (17, 18), AKT phosphorylation levels are significantly downregulated after AXL signaling is inhibited in mesothelioma or metastatic breast cancer (17, 19, 20). Although AXL, as an important oncogene, has been demonstrated to be highly expressed in human prostate cancer, leukemia, ductal carcinoma, and breast cancer among others (17, 21–23), its biological function in HCC is not well elucidated. AXL regulates tumor invasion through the transcriptional activation of SLUG in HCC (24). Reichl et al. found that AXL knockdown severely impaired resistance to TGF-β mediated growth inhibition, cell invasion, and trans-endothelial migration in HCC (23). In addition, AXL expression could be observed in liver tumor tissues and microvascular cells, but not in non-tumorous liver parenchyma (17). Furthermore, overexpression of AXL in HCC cell lines was shown to promote cell proliferation and migration, while inhibiting apoptosis (23), a study of HCC patients undergoing hepatectomy also confirmed that the patients with AXL expression showed more aggressive pathological features of tumor invasiveness and had a statistically higher tumor recurrence rate and lower survival rate 5-years after surgery (25). Increasing evidence shows that the crosstalk between tumor-derived ECs (TECs) and cancer cells promotes HCC metastasis (26). However, the biological function of AXL in microvascular cells has not yet been demonstrated. In our present study, to our surprise, AXL was highly expressed in TECs, but not in tumor cells, and was negatively associated with overall survival (OS) and disease-free survival (DFS) in HCC patients. Our new findings indicated that AXL overexpression in TECs promotes vessel metastasis in HCC patients, and thus might be a potential therapeutic target of PVTT.

## Materials and Methods

### Patients for Clinical Analysis and Follow-Up (The Data Was Used in Result 3.4)

The clinicopathological features of HCC patients who underwent R0 curative resections at the Eastern Hepatobiliary Surgery Hospital from 2003 to 2013 were retrospectively reviewed. The inclusion criteria were: (1) no preoperative or postoperative treatment, (2) no residual tumors identified on inspection and histological examination of the margins after resection, (3) PVTT confirmed by preoperative imaging and observations during the surgery and by pathological diagnosis, and (4) no severe preoperative dysfunction. Patients who could not be followed-up within two months after hospital discharge or who died due to complications were excluded from our study. The classification of the EC density (marked by CD34) was evaluated by pathologists based on the amount of positive tumor vasculature and the intensity of the staining.

The patients were followed-up every 2 to 3 months until dropout or death. If recurrence was confirmed, patients received appropriate treatments, such as transhepatic arterial chemotherapy and embolization, or liver resection, depending on the condition of the patients. The diagnosis of recurrence was obtained using ultrasound scanning, magnetic resonance imaging, computed tomography, and raised serum a-fetoprotein (AFP) levels. OS was defined as the duration from resection to the time of last follow-up or death. Recurrence-free survival (RFS) was defined as the duration from resection to the time of recurrence.

The baseline characteristics of the patients in the two groups are shown in **Table S2** (before propensity score matching (PSM) analysis) and **Table S3** (after PSM).

### Patients for Tissue Microarray and Follow-Up (The Data Was Used in Result 3.2)

A total of 305 specimens were used. All patients underwent curative resection between 2003 and 2013 at the Eastern Hepatobiliary Surgery Hospital and did not receive any anti-cancer therapy before surgery. The curative resection criteria were defined as macroscopically complete removal of the tumor as described previously (27). Tumor stage was assessed according to the 7th edition of the AJCC/UICC TNM classification system (28). Tumor differentiation was determined by the Edmondson grading system. Follow-up procedures and post-operative treatment modalities were performed according to uniform guidelines, as described previously (27). The median observation time was 32.27 months (range, 1.0 – 69.8). This study was approved by the ethics committee of the Eastern Hepatobiliary Surgery Hospital (Shanghai, China) and all patients provided their written informed consent to participate in this study. The relationship between AXL expression and clinicopathological features is shown in **Table S10**. The details of univariate and multivariate analyses are shown in **Tables S11** and **S12**.

### Cell Culture and the Collection of Conditioned Media

Human HCC cell lines, HCCL-M3 and MHCC-97L (established at our institute) were cultured in Dulbecco’s modified Eagle’s medium (Invitrogen, Carlsbad, CA, USA) supplemented with 10% fetal bovine serum (Invitrogen), 100

IU/mL penicillin G and 100 mg/mL streptomycin sulfate (Sigma-Aldrich, St. Louis, MO, USA) in a humidified atmosphere of 5% CO_2_ at 37°C.

TECs and normal ECs (NECs) were isolated from surgical HCC specimens and the surrounding normal liver tissues, as previously described (29). Primary human umbilical vein endothelial cells (HUVECs; AllCells, Shanghai, China) were cultured in endothelial cell growth medium-2 (Lonza, Basel, Switzerland) supplemented with 10% fetal bovine serum and were used within 2-10 passages.

Conditioned media (CM) for ECs was generated according to our previous study (30).

### Immunohistochemical Assay

Tumor tissues from patients and nude mice were fixed in 10% formalin and embedded in paraffin, and immunohistochemistry was performed by modification (31, 32). Briefly, 5-μm-thick tumor tissue slices were deparaffinized with xylene and rehydrated in gradient alcohol (anhydrous, 95%, and 75%). After washing three times in PBS, the endogenous peroxidase of the tissue slices was blocked by incubation with 0.3% H_2_O_2_ for 10 min. Following incubation with sodium citrate antigen retrieval solution for 10 min, the sections were incubated with blocking solution for 30 min, followed by incubation with primary antibodies against AXL (1:100; Cell Signaling Technology, 8661S, Danvers, MA, USA) and CD31 (1:100; ab182981, Abcam, Cambridge, MA, USA) overnight at 4°C. Then, the sections were incubated with secondary antibody conjugated with horseradish peroxidase (HRP) for 1 h at room temperature (RT). After DAB coloring reaction, the images were captured by light microscopy (Olympus, Japan). Positive expressions of target proteins were displayed in brown, and the cell nuclei were stained in blue with hematoxylin. In total, eight randomly selected fields were quantified with Image-Pro Plus software (Media Cybernetics, Inc).

### Immunofluorescence Staining

Tumor tissues from patients and nude mice were fixed in 4% polyformaldehyde. The immunofluorescence staining procedures were described in our previous study (30). Primary antibodies against AXL (1:100; Cell Signaling Technology, 8661S, Danvers, MA, USA) or CD31 (1:100; ab9498, Abcam) were used.

### Gene Expression Microarray

Total RNA was extracted from the TECs or NEC of four HCC patients with PVTT. Gene expression profiling was performed using the Affymetrix Human Genome U133 Plus 2.0 Array (Affymetrix, USA) at ShanghaiBio (National Engineering Center for Biochips, Shanghai, China).

### Single Cell RNA-Seq Workflow

Previous studies mainly focused on AXL expressed in tumor cells, however, whether AXL is also expressed in other cells types, has not yet been clarified. Therefore, in the present study, single-cell RNA-seq was used to assay AXL expression in different cells types, both in tumor and nonmalignant tissues. Tumor or nonmalignant tissues were dissociated into a single-cell solution as previously described (33). Briefly, tissues were cut into 1-mm^2^ pieces and digested in dissociation buffer (RPMI-1640 medium supplied with 2% FBS, 0.05 mg/ml collagenase I, 0.05 mg/ml collagenase IV, 0.025 mg/ml hyaluronidase and 0.01 mg/ml DNase I) at 37°C for 30 min. After stopping the digestion with excess RPMI-1640 medium and lysing RBCs, the cell strainer-filtered single cell solution was maintained on ice until loading to a BD rhapsody cartridge for single cell transcriptome isolation.

Raw reads for a single cell transcriptome were processed through a whole transcriptome assay analysis pipeline, which included mapping reads to the Genome Reference Consortium Human Build 38 patch release 7 (GRCh38.p7) using Star (version 2.5.2b), annotating molecules with the recursive substitution error correction (RSEC) and distribution-based error correction (DBEC) algorithms, and annotating the results in accordance with GENCODE Release 25 (GRCh38.p7). The pipeline generated gene expression matrices were corrected by RSEC and DBEC algorithms, which were used for subsequent cluster analyses. Cells that were determined as multiples were excluded from subsequent procedures.

Raw gene expression matrices were read into R (version 3.5.1) and converted to a Seurat object using the Seurat R package (version 2.3.4). Cells with more than 60% UMIs derived from the mitochondrial genome were removed. Finally, 3605 cells remained after the filtering step. The gene expression matrix was then normalized to the total cellular UMI count. Highly variable genes were selected as having log normalized average gene expressions between 0.05 and 3 and dispersion levels above 0.5. After scaling the data with respect to UMI counts, principal component analysis (PCA) was performed based on the highly variable genes identified in the previous step to reduce dimensionality. In addition, the first 15 principal components were chosen based on a PC heat map, a Jackstraw plot, and a PC elbow plot to further reduce dimensionality using the tSNE algorithm. Eleven clusters were identified with the default setting using the RunTSNE function. Each cluster was then annotated with the canonical cell markers.

To further analyze endothelial cells (ECs), cells from Cluster 3 and Cluster 8 with a positive CD31 expression were extracted. Then, the AXL expression levels for different clusters were plotted.

### AXL Transfection

Lentivirus with induced AXL expression vector, reduced AXL expression vector and negative control vector were obtained from Genechem (GeneChem, Shanghai, China) and generated in HEK 293 cells. These vectors were transfected into HEK 293-T cells using Lipofectamine 2000 (Invitrogen) and HUVECs were transfected with the virus according to the manufacturer’s instructions. We named these cells as HUVEC-AXL-OE (HUVECs with AXL overexpression) HUVEC-AXL-KD (HUVECs with AXL knockdown) and HUVEC-AXL-NC (HUVECs with empty AXL plasmid). Western blotting was performed to measure AXL expression for validation. The most effective oligonucleotides for AXL (5ʹ-GACGAAAUCCUCUAUGUCAdTdT-3ʹ, sense) were used in the study.

### siRNA and Transfection

Cells were transfected with either a nonspecific control or AXL small interfering RNA (siRNA) (Sigma-Aldrich), CCL14 siRNA (Santa Cruz, Santa Cruz, CA, USA) and Dickkopf-1 (DKK-1) siRNA (Genepharma, Shanghai, China) according to the manufacturer’s instructions. The siRNA sequences are listed in **Table S1**.

### Tube Formation Assay

As described in our previous study (30), ECs (1.5 × 10^4^/well) were added to Matrigel-coated 96-well plates (Corning, Tewksbury. MA) and incubated at 37°C for 6 h with CMs. Representative images were captured using an inverted microscope. Tube formation was assessed by measuring the length of the tube at × 200 magnification. The relevant effects of CM were normalized according to total cellular protein.

### Cell Viability Assay

Cell viability was assayed using CCK-8 solution (Dojindo, Kumamoto, Japan). After treatment, the cells were incubated with CCK-8 reagent (20 μl/well) (Cell Counting Kit-8, Dojindo, Kumamoto, Japan) and cultured at 37°C for 1 h. OD of the culture medium at 450 nm was detected by a microplate reader (Thermo, USA). Cell viability rate was calculated as follows: (absorbance of drug-treated sample/absorbance of control sample) ×100%.

### Cell Migration

Cell migration assays were performed using a chamber (Corning, Tewksbury. MA, USA) with an 8.0-μm polycarbonate filter inserted into a 24-well plate, as previously described (34). Relevant effects of CM were normalized according to total cellular protein.

### Human Cytokine Antibody Array and ELISA

The levels of secreted cytokines were compared between the CMs of KD and NC cells using a Human Cytokine Antibody Array (Human 507, AAH-BLG-1, RayBiotech, Norcross, GA, USA) according to the manufacturer’s instructions. The protein concentrations of CCL14 and DKK-1 in the supernatants were also measured using an enzyme-linked immunosorbent assay (ELISA) kit (CCL14: EK1123 Boster, Wuhan, China; DKK-1: EK0867 Boster, Wuhan, China) according to the manufacturer’s instructions.

### Western Blot Assay

Western blot assay was performed according to the procedure described in our previous study (35) with slight modification. In brief, a total of 30 μg protein from each sample was loaded and separated by standard SDS-PAGE (8%, 10% or 12%) and then transferred onto PVDF membranes by wet transfer. After blocked with 5% (w/v) BSA in PBS, the PVDF membranes were incubated with primary antibodies against AXL (1:1000; Cell Signaling Technology, 8661S, USA), PI3K p110α (1:1000; Cell Signaling Technology, 4249S, USA), p-AKT(1:500; ab38449, Abcam), pan-AKT (1:500; ab8805, Abcam), SOX2 (1:1000; Cell Signaling Technology, USA), DKK-1 (1:1000; Cell Signaling Technology, 48367S, USA), and GADPH (1:1000; Cell Signaling Technology, 5174T, USA), at 4°C overnight. Then, the PVDF membranes were incubated with horseradish peroxidase (HRP)-conjugated secondary antibodies for 1 h. After the target protein bands were visualized and captured, Image J software (National Institutes of Health) was used to assay and quantify the expression of target proteins. Target proteins were normalized to glyceraldehyde-3-phosphate dehydrogenase (GAPDH) levels within the same sample.

### HCC Cell Line Xenograft Model and Patients-Derived Xenograft (PDX) in Nude Mice

All animal experiments were performed in accordance with the protocols approved by the Animal Ethics Committee of Second Military Medical University (EHBH-KY-2018017), which complies with international rules and policies for laboratory animal use and care as founded in the European Community guidelines (EEC Directive of 1986; 86/609/EEC). For the xenograft model, 12 male BALB/c nude mice (5 weeks old) were randomized into four groups. HCC cells (approximately 5 × 10^6^ cells) mixed with ECs (approximately 1 × 10^6^ cells) in 200 μL normal saline were implanted *via* subcutaneous injection to generate subcutaneous tumors. After 1 week, the mice were euthanized, and the cancerous tissues were cut into 2 mm^3^ pieces. The xenograft HCC model was established (n = 5 in each group) by orthotopic implantation of the tissues into the liver (36). After 5 weeks, the mice were killed, the tumor volume was measured, and the tumors or lungs were fixed using a 4% paraformaldehyde solution. The tumor volume was calculated according to the following formula: (largest diameter × [perpendicular height]^2^)/2. The number of lung or liver metastases was confirmed by H & E staining.

For the PDX HCC model, fresh HCC samples obtained from clinical hepatectomies were used to establish an orthotopic model in the nude mice, as previously described (37). Briefly, HCC tissues were collected from fresh resected specimens, and subcutaneously inoculated in nude mice to generate HCC PDX models. When the tumors had grown, they were isolated and divided into tissue blocks (3 mm^3^) to generate homogeneous first generation PDX models. Once these models were mature, they could be divided into different experimental groups. Forty-eight hours after inoculation, the mice were randomly assigned to the treatment group or the control group (n = 5) and administered an oral dose of R428 (75 mg/kg twice daily; Selleckchem, Huston, TX, USA) or vehicle until day 28 (20).

### Hematoxylin Eosin (H & E) Staining

H & E staining was performed to assess the pathological changes into the tumor tissues and to measure the number of lung or liver metastases.

### Statistical Analysis

SPSS 22.0 for Windows (SPSS Inc., Chicago, IL, USA) was used for the statistical analyses in this study. Categorical variables were analyzed using the Pearson χ 2 test or the Fisher’s exact test. Quantitative variables were analyzed using ANOVA or the Student’s t test. Survival was assessed using Kaplan-Meier curves and compared using the log-rank test. Univariate and multivariate analyses were performed using the Cox proportional hazards regression model. Correlational analyses were performed using the Pearson correlation analysis. Because there was heterogeneity between the high CD34 group and low CD34 group in the study population, PSM was performed. PSM was performed on the alpha-fetoprotein (AFP) levels. P < 0.05 was considered statistically significant.

## Results

### AXL Was Mainly Expressed in TEC But Not in Tumor Cells

**Figure 1:**
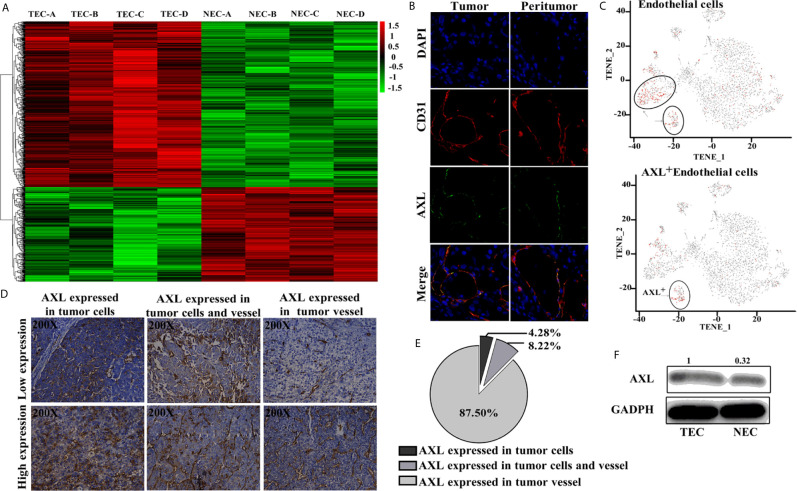
AXL is mainly expressed in TECs. **(A)** Heat map of the differentially expressed genes in the Affymetrix U133 plus 2.0 array. **(B)** AXL and CD31 expression in the ECs of HCC samples. **(C)** AXL^+^ ECs represent a subgroup of *CD31* ^+^ ECs. **(D)** AXL expression in the vessels was present in 87.5% of the total samples. **(E)** AXL expression in the HCC cells was present in only 4.28% of the samples; AXL expression in both vessels and HCC cells was present in 8.22% of the samples. **(F)** AXL was expressed at higher levels in the TECs compared with the normal ECs.

As shown in **Figure 1A**, results from the Affymetrix U133 plus 2.0 array demonstrated that, compared with the NECs, there was a completely different mRNA profile in HCC TECs (**Figure 1A**). A total of 2,590 differentially expressed genes with a two-fold change were identified, and 413 genes were shown to have a three-fold change. *AXL* was one of seven genes with the greatest fold-change and was higher in TECs compared with NECs in our array. Furthermore, *AXL* was co-expressed with *CD31*, both in human HCC tumor tissue and peritumor tissues (**Figure 1B**). The results of single-cell mRNA sequencing revealed that *AXL* was mainly expressed in ECs, and that *AXL* ^+^ *CD31* ^+^ ECs were a subgroup of *CD31* ^+^ ECs (**Figure 1C**). As demonstrated in **Figures 1D, E**, immunofluorescence staining of sections from human HCC tissues revealed that AXL expression was primarily located on tumor vessels expressing CD31. Indeed, AXL was expressed in tumor vessels (87.5%, n = 266), in HCC tumor cells (4.28%, n = 13), and in both HCC tumor cells and TECs (8.22%, n = 25) of total samples. AXL expression in the TECs was higher than that in the NECs (**Figure 1F**).

### High Expression of AXL in TECs Was Associated With the Poor OS and DFS of HCC Patients With PVTT

**Figure 2:**
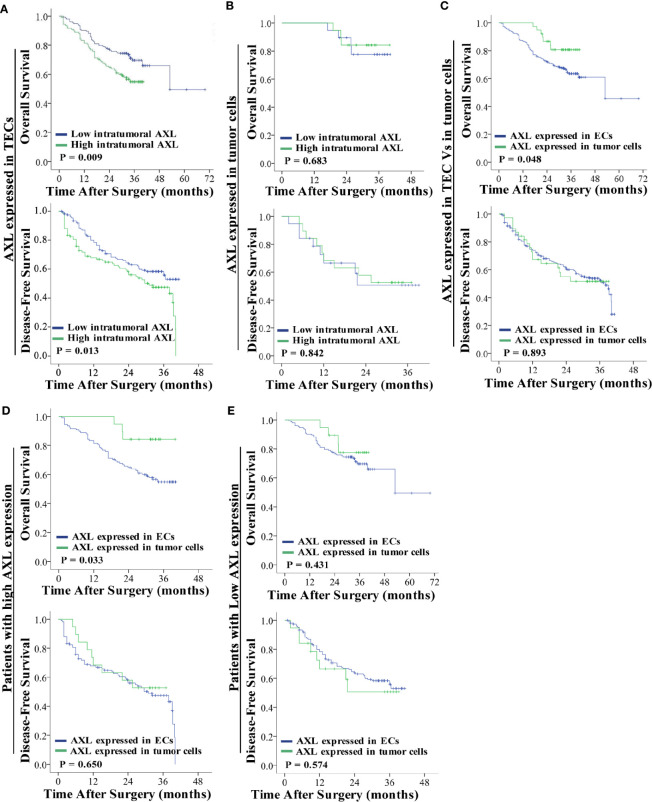
AXL overexpression in TECs but not tumor cells indicates poor prognosis. **(A)** Low expression of AXL in TECs was associated with prolonged survival (OS: p = 0.009; DFS: p = 0.013). **(B)** AXL overexpression in tumor cells indicated no significant association with poor prognosis. **(C)** AXL expressed in TECs was associated with lower OS compared with AXL expressed in TCs. **(D)** High expression of AXL in TECs indicated poorer survival compared with high expression of AXL in TCs (OS: p = 0.033; DFS: p =0.650). **(E)** There was no significant difference in survival between patients with low expression of AXL in TECs and patients with low expression of AXL in TCs (OS: p = 0.431; DFS: p =0.574).

As shown in **Figure 2A**, high AXL expression in TECs was associated with the poor OS (p = 0.009) and DFS (p = 0.013) of HCC patients with PVTT (**Figure 2A**). However, AXL expression in tumor cells was not correlated with OS (p = 0.683) and DFS (p = 0.842) in these patients (**Figure 2B**). Furthermore, the patients with AXL expression in TECs had lower OS (p = 0.048), but not DFS (p = 0.893), compared with the patients with AXL expression in tumor cells (**Figure 2C**). We also found that the patients with high AXL expression in TECs also had lower OS (p = 0.033) compared with those with high AXL expression in tumor cells (**Figure 2D**). There was no significant difference in OS and DFS between patients with low AXL expression in TECs and patients with low AXL expression in tumor cells (**Figure 2E**).

### AXL Promoted Cell Proliferation, Tube Formation, and Migration of ECs

**Figure 3:**
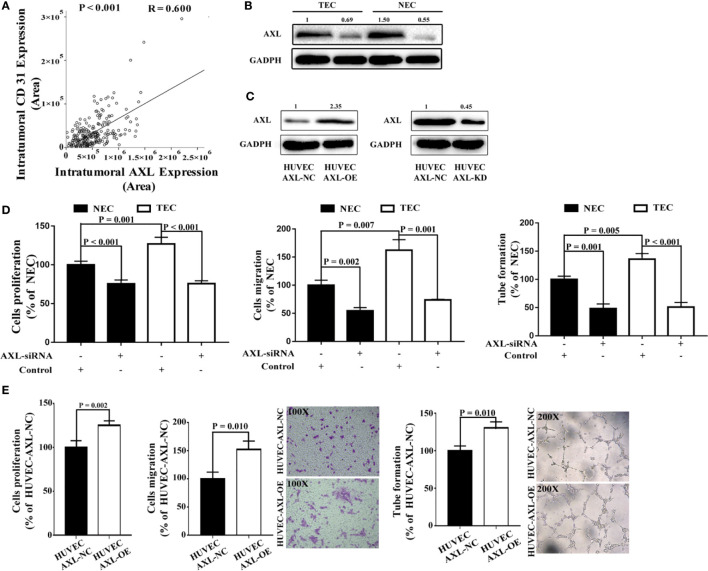
AXL expression in ECs is involved in cell migration, proliferation, and tube formation. **(A)** Intratumoral AXL expression was positively correlated with CD31 expression (p < 0.001). **(B)** AXL expression was silenced by AXLsiRNA in both NECs and TECs, as measured by western blotting assay. **(C)** AXL overexpression was induced by AXL-overexpressing plasmid, both in NECs and TECs, as measured by western blotting assay. **(D)** AXL siRNA decreased the cell proliferation (p < 0.001), migration (NEC: p = 0.002; TEC: p = 0.001), and tube formation ability (NEC: p = 0.001; TEC: p < 0.001) of both NECs and TECs. **(E)** Overexpression of AXL in HUVECs increased the cell proliferation (p = 0.002), migration (p = 0.010), and tube formation (p = 0.010) ability of HUVECs.

To access whether AXL expression in ECs may promote angiogenesis in tissues of HCC patients, we examine the expression of CD31 in the tissue microarray and analysis the relation between AXL expression and CD31 expression. As shown in **Figure 3A**, there was a positive relationship between CD31 expression and AXL expression in tumor cells (R = 0.600, p < 0.001), which indicates that AXL might promote angiogenesis in the tumors of HCC patients with PVTT. To further study the function of AXL in endothelial cells, AXL expression was silenced by AXL siRNA both in NECs and TECs (**Figure 3B**). Moreover, AXL was overexpressed or knocked down by plasmid transfection in HUVECs (**Figure 3C**). As demonstrated in **Figure 3D** and **Figure S1A**, the cell proliferation, migration, and tube formation of NECs and TECs were significantly decreased by AXL siRNA. Moreover, the cell proliferation, cell migration, and tube formation of HUVECs were greatly increased by AXL overexpression (**Figure 3E**). As expected, the cell proliferation, migration, and tube formation of HUVECs were significantly decreased by AXL knock down (**Figure S1B**).

### High Expression of AXL in ECs Promoted Cell Migration but Not Proliferation of HCC Cells

**Figure 4:**
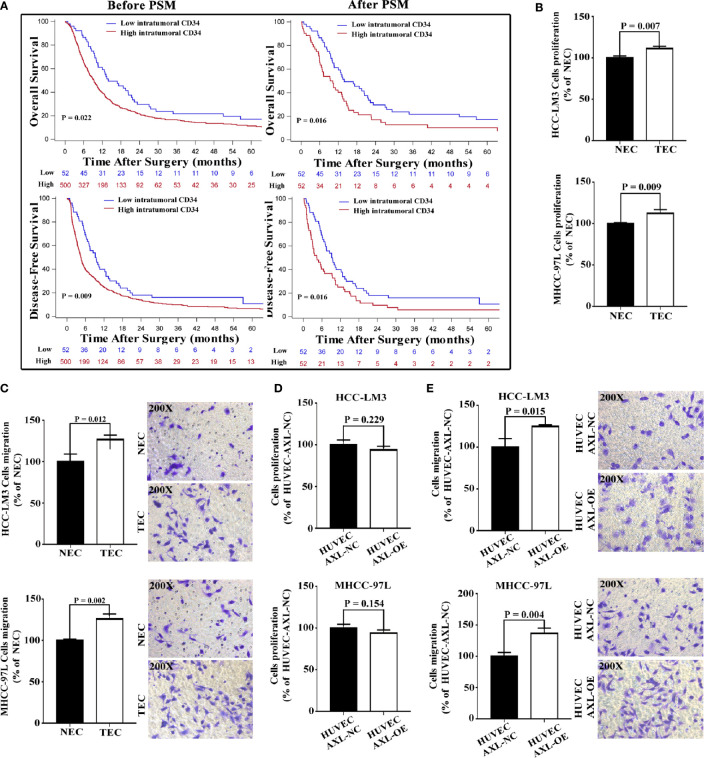
Overexpression of AXL in HUVECs promotes migration but not proliferation of HCC cells. **(A)** Before PSM, high intratumoral CD34 expression was shown to be associated with poor OS and DFS in HCC patients with PVTT (PVTT: p = 0.022 and p = 0.009). After PSM, high intratumoral CD34 expression was shown to be associated with poor OS and DFS in HCC patients with PVTT (PVTT: p = 0.016 and p = 0.016). **(B)** The culture medium of TECs promoted the proliferation of HCC-LM3 cells and MHCC-97L cells compared with the culture medium of NECs. **(C)** The culture medium of TECs promoted the migration of HCC-LM3 cells and MHCC-97L cells compared with the culture medium of NECs. **(D)** The culture medium of HUVECs overexpressing AXL could not promote the proliferation of HCC-LM3 cells and MHCC-97L cells compared with that of normal HUVECs. **(E)** The culture medium of HUVECs overexpressing AXL promoted the migration of HCC-LM3 cells and MHCC-97L cells compared with that of normal HUVECs.

TECs not only participate in the formation of vessels and provide channels for HCC metastasis, but they also promote the migration of HCC cells. EC density may be related to metastasis and the poor survival of HCC patients. We first analyzed the relationship between intratumoral EC density (marked by CD34) and the presence of PVTT (**Figure 4A** and **Table S8**). We collected data on HCC patients who were diagnosed with HCC with PVTT (n = 552) at the Eastern Hepatobiliary Surgery Hospital from 2003 to 2013. The results revealed that the number of patients with PVTT in the high EC density group was much higher than that in the low EC density group (90.58% vs 9.42%, p < 0.001). In addition, high EC density was associated with poor OS and DFS in HCC patients with PVTT (p = 0.022 and p = 0.009, respectively).

To eliminate the effect of clinicopathological features such as HBeAg, AFP, and cirrhosis on the comparison between the high and low EC density groups, PSM was used. After PSM, similar results were obtained (PVTT: p = 0.016 for OS, and p = 0.016 for DFS). These results indicated that high intratumoral EC density was associated with the presence of PVTT and the poor survival of HCC patients with PVTT.

As demonstrated in **Figures 4B, C**, compared with CM from NECs, CM from TECs significantly increased the cell proliferation and migration of HCC cells (HCC-LM3 cells and MHCC-97L cells). However, CM from HUVEC-AXL-OE cells did not increase the cell proliferation of these HCC cells compared with CM from HUVEC-AXL NC cells (**Figure 4D**). Also, CM from HUVEC-AXL-OE cells increased the migration of HCC cells compared with CM from HUVEC-AXL-NC cells (**Figure 4E**). As expected, CM from HUVEC-AXL-KD cells decreased the migration of HCC cells, but showed no effect on their proliferation compared with CM from HUVEC-AXL-NC cells (**Figure S2**).

### AXL Promoted HCC Migration, EC Migration, and Tube Formation Through the Activation of PI3K/AKT/SOX2/DKK-1 Axis

**Figure 5:**
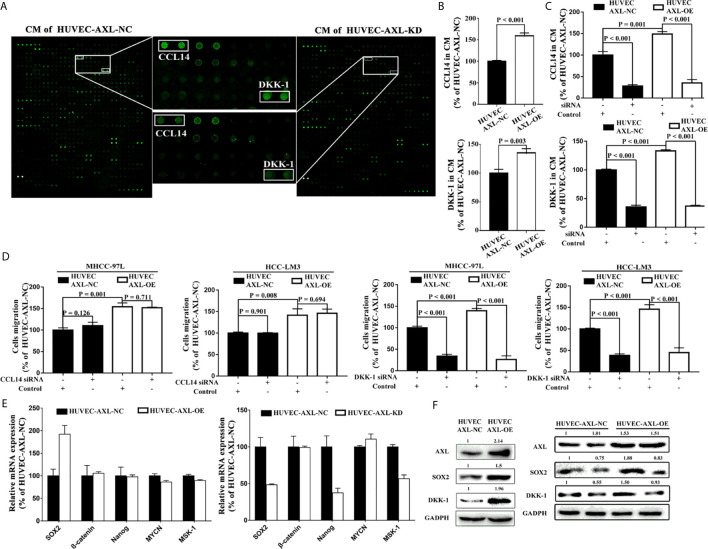
AXL/SOX2/DKK-1 axis in HUVECs promotes HCC metastasis. **(A)** DKK-1 and CCL14 secretion was significantly downregulated in CM from HUVEC-AXL-KD compared with that from HUVEC-AXL-NC, as detected with a human cytokine antibody array. **(B)** DKK-1 and CCL14 expression was markedly upregulated in the CM of HUVECs overexpressing AXL (CCL14: p < 0.001; DKK-1: p = 0.003) compared with the CM of HUVEC-AXL-NC, as detected by ELISA assay. **(C)** *AXL* siRNA downregulated DKK-1 and CCL14 secretion in the CM of HUVEC-AXL-NC and HUVEC-AXL-OE cells (CCL14: p < 0.001 and p < 0.001; DKK-1: p < 0.001 and p < 0.001). **(D)** *DKK1* siRNA (MHCC-97L: p < 0.001 and p < 0.001; HCC-LM3: p <0.001 and p < 0.001), but not *CCL14* siRNA (MHCC-97L: p = 0.126 and p = 0.711; HCC-LM3: p = 0.901 and p = 0.694) could attenuate the effect of the CM from HUVEC-AXL-NC and HUVEC-AXL-OE cells on the migration of HCC-LM3 cells and MHCC-97L cells. **(E)** *SOX2* mRNA expression was significantly increased in HUVEC-AXL-OE cells and decreased in HUVEC-AXL-KD cells compared with HUVEC-AXL-NC cells (HUVEC-AXL-KD: p < 0.001, HUVEC-AXL-OE: p < 0.001). **(F)** AXL overexpression could significantly increase SOX2 and DKK-1 protein expression in HUVEC-AXL-OE cells compared with HUVEC-AXL-NC cells, and *SOX2* siRNA inhibited SOX2 and DKK-1 protein expression in HUVEC-AXL-OE and HUVEC-AXL-NC cells.

To further investigate how AXL promoted the migration of HCC cells, a human cytokine antibody array was used. Results from CM of HUVEC-AXL-NC cells and HUVEC-AXL-KD cells showed that the secretion of CCL14 and DKK-1 was significantly downregulated in the CM of HUVEC-AXL-KD cells compared with the CM of HUVEC-AXL-NC cells (**Figure 5A**). Similarly, AXL overexpression significantly upregulated the secretion of both CCL14 and DKK-1, compared with that in HUVEC-AXL-NC cells (**Figure 5B**). As expected, AXL siRNA could counteract the secretion of CCL14 and DKK-1 in the CM of both HUVEC-AXL-NC cells and HUVEC-AXL-OE cells (**Figure 5C**). Furthermore, the increased cell migration of HCC cells induced by the CM of HUVEC-AXL-OE cells could be abolished by DKK1 siRNA but not CCL14 siRNA. Similar results were obtained in HUVECs-AXL-NC cells (**Figure 5D**).

To detect which transcription factor promoted DKK-1 expression induced by AXL, we further quantified the mRNA expression of five transcription factors that regulate DKK-1 secretion in HCC cells (38). Compared with HUVEC-AXL-NC cells, SOX2 mRNA expression was significantly upregulated in HUVEC-AXL-OE cells and downregulated in HUVEC-AXL-KD cells (**Figure 5E**). Meanwhile, compared with HUVEC-AXL-NC cells, the protein expression of AXL, SOX2, and DKK-1 was enhanced in HUVEC-AXL-OE cells. SOX2 siRNA abolished the increased protein expression of SOX2 and DKK-1 in HUVEC-AXL-OE cells (**Figure 5F**). Similar results were obtained in HUVEC-AXL-KD cells (**Figure S3**).

**Figure 6:**
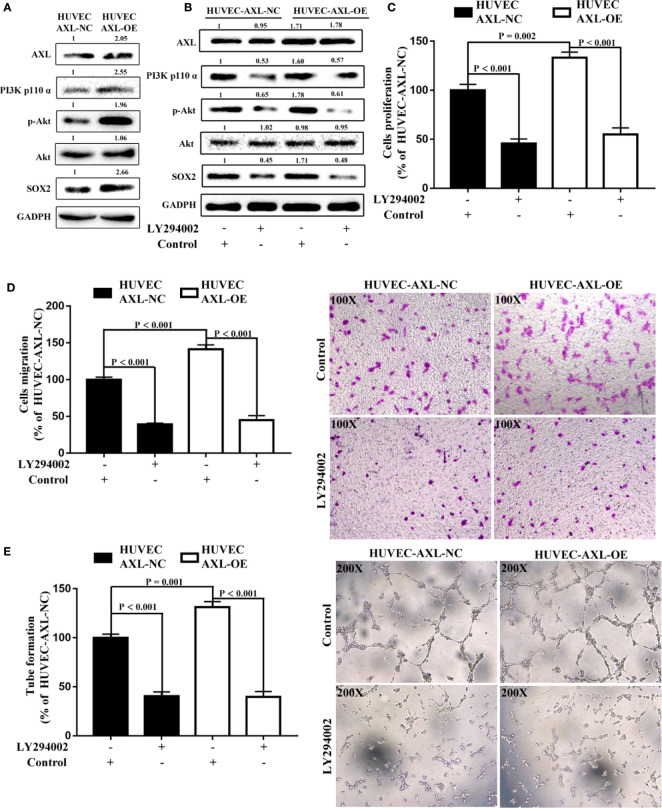
AXL/SOX2/DKK-1 axis modulates the cell migration and tube formation of HUVECs through overactivation of the PI3K/AKT signaling pathway. **(A)** The expression levels of the PI3K/AKT signaling pathway were higher in HUVEC-AXL-OE cells compared with HUVEC-AXL-NC cells. **(B)** LY29400 downregulated the expression levels of the PI3K/AKT signaling pathway in HUVEC-AXL-NC and HUVEC-AXL-OE cells. **(C–E)** LY294002 inhibited cell proliferation (NC: p < 0.001; OE: p < 0.001), migration (HUVEC-AXL-NC: p < 0.001; HUVEC-AXL-OE: p < 0.001), and tube formation (HUVEC-AXL-NC: p < 0.001; HUVEC-AXL-OE: p < 0.001).

It has been reported that the PI3K/AKT signaling pathway plays a critical role in tumor angiogenesis and can be modulated by AXL (17, 19, 20, 39). In the present study, compared with HUVEC-AXL-NC cells, the protein expression of AXL, PI3K p110α, p-AKT, and SOX2 was enhanced in HUVEC-AXL-OE cells (**Figure 6A**). LY294002, a PI3K inhibitor, could abolish the increased protein expression induced by AXL (**Figure 6B**) and showed no obvious effect on AXL protein expression in HUVEC-AXL-OE cells. The PI3K/AKT pathway is downstream of AXL but upstream of the SOX2/DKK-1 axis17-18. In a further study, LY294002 could also decrease the cell proliferation, migration, and tube formation of HUVEC AXL-NC cells and HUVEC AXL-OE cells (**Figures 6D, E**). Similar results were obtained in HUVEC-AXL-KD cells (**Figure S4**). These results indicate that AXL modulated HCC migration through the SOX2/DKK-1 axis, as well as the proliferation, migration, and tube formation of ECs by regulating the PI3K/AKT/SOX2/DKK-1 axis.

### AXL in ECs Promoted Tumor Growth and Metastasis of HCC *In Vivo*

**Figure 7:**
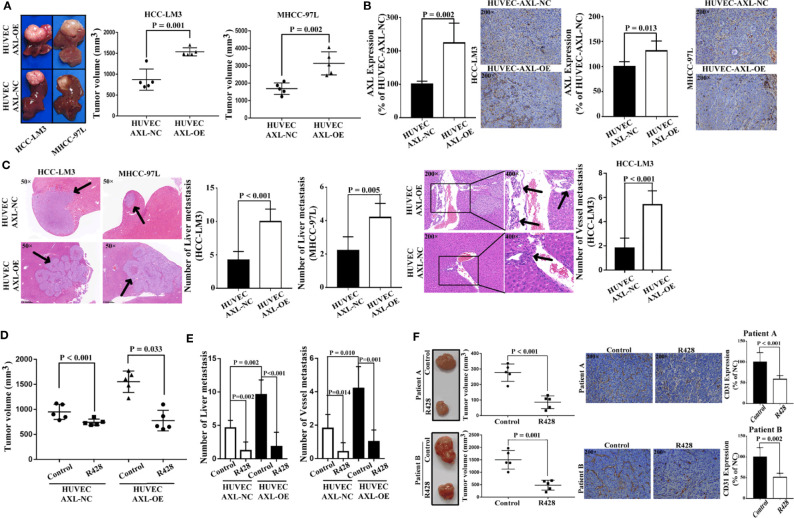
HUVECs overexpressing AXL promote tumor growth and metastasis of HCC *in vivo*. **(A)** HUVECs overexpressing AXL promoted tumor growth *in vivo* (HCC-LM3: p = 0.001; MHCC-97L: p = 0.002). **(B)** AXL was highly expressed in the vessels of the HUVEC-AXL-OE group compared with the HUVEC-AXL-NC group (HCC-LM3: p = 0.002; MHCC-97L: p = 0.013). **(C)** HUVECs overexpressing AXL promoted vessel metastasis and liver metastasis of HCC-LM3 cells *in vivo*, and HUVECs overexpressing AXL, but not HUVEC-AXL-NC cells, induced liver metastasis and vessel metastasis of MHCC-97L cells *in vivo*. **(D)** R428 treatment decreased tumor volume induced by HUVECs overexpressing AXL and HCC-LM3 cells or MHCC-97L cells. **(E)** R428 treatment decreased vessel metastasis and MVI of HCC induced by HUVECs overexpressing AXL and HCC-LM3 cells or MHCC-97L cells. **(F)** R428 treatment decreased tumor volume in the HCC PDX models (patient A: p < 0.001; patient B: p = 0.001). Furthermore, in the HCC PDX models, CD31 expression in the tumors of mice treated with R428 was lower compared with that in the tumors of mice treated with the control (patient A: p < 0.001; patient B: p = 0.002).

As shown in **Figure 7A**, HUVEC AXL-OE cells significantly increased the tumor volume of xenograft nude mice subcutaneously inoculated with HCC-LM3 cells and MHCC-97L cells *in vivo*. As expected, compared with HUVEC cells, HUVEC AXL-OE cells significantly increased AXL expression in the tumor tissue of xenograft mice subcutaneously inoculated with HCC-LM3 cells and MHCC-97L cells *in vivo* (**Figure 7B**). Furthermore, compared with HUVEC-AXL-NC cells, HUVEC AXL-OE cells resulted in more liver metastasis of MHCC-97L cells *in vivo*. In addition, HUVEC AXL-OE cells induced liver metastasis and vessel metastasis of HCC-LM3 cells *in vivo* compared with HUVEC-AXL-NC cells (**Figure 7C**). R428, an inhibitor of AXL, significantly abolished the increased tumor volume (**Figure 7D**), liver metastasis, and vessel metastasis in the xenograft nude mice inoculated subcutaneously with HCC-LM3 cells plus HUVEC-AXL-OE or HUVEC-AXL-NC cells (**Figure 7E**). Similarly, in the PDX model, R428 significantly reduced the tumor volume and CD31 protein expression in HCC tumor tissues (**Figure 7F**). Analogous results were obtained in HUVECs with AXL knock down (**Figure S5**).

## Discussion

In clear cell renal cell carcinoma (ccRCC), AXL expression is proved to be positively related with anti-angiogenic drug resistance and poor survival. And AXL inhibitor cabozantinib or an ultra-high affinity soluble AXL Fc fusion decoy receptor (sAXL) inhibited the growth of a pazopanib-resistant ccRCC patient-derived xenograft. Further study demonstrated that GAS6/AXL signaling activated S100A10 expression through SRC to promote ccRCC angiogenesis and endothelial cell invasion (40). AXL has been also shown to have a critical role in the progression and metastasis of HCC. However, epithelial HCC cell lines fail to release soluble AXL, while mesenchymal HCC cell lines release high levels of soluble AXL and liver myofibroblasts release soluble AXL (16, 23), which indicates that non-epithelial HCC cells might contribute mainly to AXL expression. Axl expression on endothelial cells is involved in regulating normal and tumor vasculature (41, 42). However, the complex function of AXL in angiogenesis under normal or pathological conditions especially in TECs needs to be further addressed. In present study, we found that AXL was mainly expressed in the TECs but not in the tumor cells of HCC patients. Furthermore, TECs expressed higher levels of AXL than NECs. AXL in ECs can promote angiogenesis, and high intratumoral EC density was positively associated with the poor OS and DFS of HCC patients with PVTT (**Figure 4A** and **Tables S4–S7**), which indicates that high AXL expressed by TECs contributed to the poor OS and DFS of HCC patients with PVTT.

In the present study, HCC patients with high expression of AXL in TECs had lower OS and DFS compared with patients with lower expression of AXL in TECs. However, high expression of AXL in the tumor cells of HCC patients did not affect OS and DFS, compared with patients with low AXL expression in tumor cells, which indicates that AXL expression in TECs played a crucial role in promoting tumor progression and metastasis. Further results demonstrated that AXL overexpression contributed to the cell proliferation, migration, and tube formation of TECs and NECs. CM of TECs isolated from tumor tissues of HCC patients with PVTT could significantly increase the cell proliferation and migration of HCC cells. However, CM of HUVECs overexpressing AXL could enhance the migration of HCC cells, but showed no obvious effect on the proliferation of HCC cells, which indicates the important ability of AXL in TECs on HCC metastasis, but the limited activity of AXL on HCC tumor growth.

SOX2, as an embryonic stem cell-specific transcription factor, is a critical factor controlling the self-renewal, pluripotency, and proliferation of embryonic stem cells (43–45), and has been shown to function as an oncogene in cervical carcinogenesis by promoting cell proliferation and tumorigenicity (46). High expression of SOX2 has been found in glioblastoma, breast, and cervical CSC populations (45, 47–50). DKK-1 is typically considered as an antagonist for the Wnt signaling pathway, which regulates the differentiation, proliferation, and migration of cancer cells (51). However, recent studies showed that DKK-1 might function through a non-classical Wnt signaling pathway, and the expression of DKK-1 may differ according to the cancer type. This phenomenon may explain why DKK-1 plays a dual role in tumorigenicity either as a tumor suppressor or promoter (38). In HCC, DKK-1 was shown to be abnormally overexpressed in tumor tissues or serum and was associated with multiple metastatic lymph nodes, vein infiltration, and the diagnosis of HCC (52–54). In addition, DKK-1 regulates the invasiveness and metastasis of HCC cells and can be modulated by a variety of regulatory molecules, such as p53, 1α,25-dihydroxyvitamin D3, Msh homeobox 1 (MSX), progesterone, β-catenin/TCF, and c-myc (36, 38, 52, 55). A previous study showed that AXL regulates tumor invasion through the transcriptional activation of SLUG in HCC cells (24). However, in the present study, secretion of DKK-1 was closely modulated by AXL in ECs, which in turn promoted HCC cell migration. Furthermore, SOX-2 was shown to positively regulate the expression of DKK-1 in HUVEC cells with AXL overexpression, which indicates that AXL enhances cell migration of HCC through the SOX2/DKK-1 axis. It has been reported that the PI3K/AKT signaling pathway has a critical role in tumor angiogenesis and can be modulated by AXL (17, 19, 20, 39). A further study demonstrated that AXL indeed regulated the activation of the PI3K/AKT pathway in HUVECs, and that the PI3K/AKT pathway was involved in the AXL-mediated SOX2/DKK-1 axis. Therefore, AXL promotes cell migration and tube formation of HUVECs through the activation of the PI3K/AKT/SOX2/DKK-1 axis.

After co-implantation with HCC cells in xenograft nude mice and patient-derived xenograft (PDX) nude mice, HUVECs overexpressing AXL could significantly enhance tumor growth, liver metastasis, and vessel metastasis of HCC, which could be abolished by R428, an AXL inhibitor.

Metabolic reprogramming is especially important for cancer progression and metastasis (56–58). Transglutaminase-2 can facilitate extracellular vesicle-mediated establishment of the metastatic niche (56), while pyruvate carboxylase enhances the pulmonary tropism of metastasis in breast cancer (57). Pyruvate carboxylase inhibition by 1α,25-dihydroxyvitamin D enhances oxidative stress in early breast cancer progression (58). However, whether AXL overexpression in TECs promotes vessel metastasis in patients with HCC through modulating cancer metabolism has not yet been clarified.

Our current study has some limitations. First, we did not further identify the effect of AXL in ECs on PVTT cells. Second, the detailed molecular mechanism of DKK-1 in promoting PVTT of HCC could not be addressed clearly.

In conclusion, AXL, which is mainly expressed in TECs of HCC, could promote vessel metastasis of HCC through the activation of the PI3K/AKT/SOX2/DKK-1 axis. Targeting AXL in TECs could be a potential therapeutic strategy for HCC with PVTT.

## Data Availability Statement

The datasets presented in this study can be found in online repositories. The names of the repository/repositories and accession number(s) can be found below: (https://www.biosino.org/node/review/detail/OEV000125?code=IXU5WR6Q and the Usershare data is:OEP001082, OEX009952, OEX009994, OES042489, OES042490, OES042595, OER062763, OER062764, OER062855, OED165629, OED165630, OED165631, OED165632, OED166145, OED166146.

## Ethics Statement

The studies involving human participants were reviewed and approved by The Ethics Committee of the Eastern Hepatobiliary Surgery Hospital (Shanghai, China). The patients/participants provided their written informed consent to participate in this study. The animal study was reviewed and approved by The Animal Ethics Committee of Second Military Medical University.

## Author Contributions

Conception and design: S-QC, H-CS, Z-TC, X-PZ, and J-YA. Experiments: Z-TC, X-PZ, J-YA, and X-DZ. Financial support: S-QC, M-CW, and Z-TC. Provision of study materials or patients: X-DZ and X-PZ. Data analysis and interpretation: Z-TC, X-PZ, and WL; All authors contributed to the article and approved the submitted version.

## Funding

This study was supported by the National Natural Science Foundation of China (no. 81702335) and the National Key Basic Research Program “973 project” (no. 2015CB554000).

## Conflict of Interest

The authors declare that the research was conducted in the absence of any commercial or financial relationships that could be construed as a potential conflict of interest.
