# Supplementary material for: AXL Overexpression in Tumor-Derived Endothelial Cells Promotes Vessel Metastasis in Patients With Hepatocellular Carcinoma
Source: Front Oncol. 2021 May 27;11:650963. doi: 10.3389/fonc.2021.650963 (PMC8191462; doi:10.3389/fonc.2021.650963)
Supplement: Supplementary file 1 [file DataSheet_1.pdf]

Catalogue

Supplementary figure legend 2
Supplementary tables: 5
Table S1: the sequences of siRNA 5
Table S2: the clinicopathological features of patients in the study before PSM 6
Table S3: the clinicopathological features of patients in the study after PSM 8
Table S4: Univariate analyses of factors associated with survival and recurrence for HCC patients with PVTT before PSM 10
Table S5: Multivariate analyses of factors associated with overall survival and recurrence for HCC patients with PVTT before PSM 11
Table S6 Univariate analyses of factors associated with survival and recurrence for HCC patients with PVTT after PSM 12
Table S7: Multivariate analyses of factors associated with overall survival and recurrence for HCC patients with PVTT after PSM 13
Table S8: the relational between ECs density and the PVTT in HCC patients 14
Table S9: The Cumulative DFS and OS rate of HCC patients with PVTT before PSM and after PSM in the high ECs density and low ECs density groups 15
Table S10: Relationships between intratumoral AXL expression in EC and clinicopathological features 16
Table S11: Univariate analyses of factors associated with survival and recurrence 18
Table S12: Multivariate analyses of factors associated with overall survival and recurrence 19
Supplementary Materials and Methods 20
Patients for clinical analysis and follow-up 20
Patients for tissue microarray and follow-up 20
AXL transfection 21
siRNA and transfection 21
EC tube formatting assays 21
Proliferation assay and cell migration 22
Single cell RNA-seq workflow 22
Statistical analysis 23
Supplementary Results 24
Patient characteristics 24
Risk factors for poor RFS and OS 24
Survival analysis 25
References: 25

Supplementary figure legend

Figure S1. AXL expression in ECs involved in cell migration, proliferation and tube formation of ECs. **A:** AXL siRNA decreased cell migration and tube formation ability of both NECs and TECs. **B:** AXL knockdown in HUVECs decreased cell proliferation ($P < 0.001$), migration ($P = 0.014$) and tube formation ($P = 0.002$) ability of HUVECs.

Figure S2. AXL knockdown in HUVECs decreased migration but not proliferation of HCC cells. **A:** CM of HUVEC-AXL-KD could not promote cell proliferation of HCC-LM3 cells ($P = 0.682$) and MHCC-97L cells ($P = 0.179$), compared to that of HUVEC-AXL-NC. **B:** CM of HUVEC-AXL-KD decreased cell migration of HCC-LM3 cells ($P = 0.005$) and MHCC-97L cells ($P = 0.03$), compared to that of HUVEC-AXL-NC.

Figure S3. AXL/SOX2/DKK-1 axis in HUVECs promoted HCC metastasis. **A:** Expression of DKK-1 and CCL14 were greatly down-regulated in CM of HUVEC-AXL-KD (CCL14: $P < 0.001$; DKK-1: $P < 0.001$) compared with CM of HUVECs-AXL-NC. **B:** DKK siRNA and CCL14 siRNA down-regulated DKK-1 and CCL14 secretion in CM of the HUVEC-AXL-NC and HUVEC-AXL-KD cells (CCL14: $P < 0.001$ and $P < 0.001$; DKK-1: $P < 0.001$ and $P < 0.001$). **C:** AXL expression after AXL siRNA treatment. **D:** DKK-1 siRNA (MHCC-97L: $P < 0.001$ and $P = 0.001$; HCC-LM3: $P < 0.001$ and $P = 0.005$) but not CCL14 siRNA (MHCC-97L: $P = 0.296$ and $P = 0.130$; HCC-LM3: $P = 0.245$ and $P = 0.316$) could decrease the effect of CM from HUVEC-AXL-NC and HUVEC-AXL-KD cells on the migration of HCC-LM3 cells and MHCC-97L cells. **E:** DKK-1 expression after DKK-1 siRNA treatment. **F:** DKK-1 but not CCL14 mediated the migration promoting effect of AXL on HCC-LM3 cells and MHCC-97L cells. **G:** AXL knockdown could significantly decrease the protein

expression of SOX2 and DKK-1 in HUVEC-AXL-KD cells compared with HUVEC-AXL-NC cells, and SOX2 siRNA inhibited protein expression of SOX2 and DKK-1 in HUVEC-AXL-KD and HUVEC-AXL-NC cells. **H:** Secretion of DKK-1 were greatly down-regulated both in CM of HUVEC-AXL-KD ($P < 0.001$ and $P = 0.002$) and CM of HUVECs-AXL-OE compare with that of control ($P < 0.001$ and $P < 0.001$).

Figure S4. AXL/SOX2/DKK-1 axis modulated cell migration and tube formation of HUVECs through over-activating PI3K/Akt signal pathway. **A:** PI3K/Akt signal pathway was inhibited in HUVEC-AXL-KD cells compared with HUVEC-AXL-NC cells. **B:** LY29400 downregulated the expression levels of the PI3K/Akt signaling pathway in HUVEC-AXL-NC and HUVEC-AXL-KD cells. **C-E:** LY294002 inhibits proliferation (HUVEC-AXL-NC: $P < 0.001$; HUVEC-AXL-KD: $P = 0.002$), migration (HUVEC-AXL-NC: $P < 0.001$; HUVEC-AXL-KD: $P = 0.001$) and tube formation (HUVEC-AXL-NC: $P < 0.001$; HUVEC-AXL-KD: $P = 0.010$).

Figure S5. HUVECs with AXL knockdown decreased tumor growth and metastasis of HCC *in vivo*. **A:** Mice with HUVEC-AXL-KD showed smaller tumor volume compare with that of HVUEC-AXL-NC (HCC-LM3: $P = 0.010$; MHCC-97L: $P = 0.013$). **B:** Tumor weight. **C:** AXL expression was decreased in tumors of the HUVEC-AXL-KD group compared to that of HUVEC-AXL-NC group (HCC-LM3: $P = 0.008$; MHCC-97L: $P = 0.002$). **D,** Mice with HUVEC-AXL-KD had less liver metastasis compare with that of HVUEC-AXL-NC (HCC-LM3: $P = 0.024$; MHCC-97L: $P = 0.014$). **E:** The images of vessel metastasis in mice of MHCC-97L. **F:** For HCC-LM3, the mice with HUVEC-AXL-KD showed less vessel metastasis compare with that of HUVEC-AXL-NC ($P = 0.025$). For MHCC-97L, there were no vessel metastasis found. **G:** R428

treatment decreased tumor volumes induced by HUVECs-AXL-OE in the mice with HCC-LM3 cells. **H:** R428 treatment reduced live metastasis and vessel metastasis for mice with HCC-LM3 cells. **I:** The effect of R428 on body weight. **J:** The effect of R428 on tumor weight.

Supplementary tables:

Table S1: the sequences of siRNA

siRNA	sense（5'-3'）
**AXL**	GACGAAAUCCUCUAUGUCAdTdT
**SOX2**	CCAUGGAUUUAUUCCUAAATT
**DKK-1**	GGCUCUCAUGGACUAGAAAtt
**CCL14**	AGCGGAUUAUGGAUUACUAtt

Table S2: the clinicopathological features of patients in the study before PSM

Variables	HCC patients with PVTT		
	Low ECs density (n=52)	High ECs density (n=500)	P
Age	50.31±9.867	48.76±10.423	0.471
Sex			0.842
Male	48 (92.31%)	452 (90.40%)	
Female	4 (7.69%)	48 (9.60%)	
HBeAg			0.562
Negative	40 (76.92%)	366 (73.20%)	
Positive	12 (23.08%)	134 (26.80%)	
HBV DNA			0.672
$\leq 10^4$	34 (65.38%)	312 (62.40%)	
$>10^4$	18 (34.62%)	188 (37.60%)	
AFP (ng/dL)			<0.001
≤200	19 (36.54%)	70 (14.00%)	
200-400	15 (28.85%)	97 (19.40%)	
⩾400	18 (34.62%)	333 (66.60%)	
ALT (U/L)			0.317
≤44	22 (42.31%)	248 (49.60%)	
>44	30 (57.69%)	252 (50.40%)	
TBIL	16.23±6.697	16.20±12.575	0.732
ALB(g/L)			0.977
≤35	50 (96.15%)	475 (95.00%)	
>35	2 (3.85%)	25 (5.00%)	
PT			0.537
>13	10 (19.23%)	115 (23.00%)	
≤13	42 (80.77%)	385 (77.00%)	
PLT			0.479
>100	40 (76.92%)	405 (81.00%)	
≤100	12 (23.08%)	95 (19.00%)	
Tumor diameter (cm)			0.175
≤5	6 (11.54%)	96 (19.20%)	
>5	46 (88.46%)	404 (80.80%)	
No of tumors			0.880
Solitary	49 (94.23%)	463 (92.60%)	
Multiple	3 (5.77%)	37 (7.40%)	
Tumor capsule			0.937
Complete	33 (63.46%)	306 (61.20%)	
Incomplete	6 (11.54%)	65 (13.00%)	
Absent	13 (25.00%)	129 (25.80%)	
Cirrhosis			0.437

	HCC patients with PVTT		
Variables	Low ECs density (n=52)	High ECs density (n=500)	P
No	17 (32.69%)	138 (27.60%)	
Yes	35 (67.31%)	362 (72.40%)	
BCLC			0.936
0	1 (1.92%)	12 (2.40%)	
A	48 (92.31%)	454 (90.80%)	
B	3 (5.77%)	34 (6.80%)	
Child Pugh			0.413
A	52 (100.00%)	485 (97.00%)	
B	0 (0.00%)	15 (3.00%)	
C	0 (0.00%)	0 (0.00%)	

Abbreviation：AFP: alpha fetoprotein, ALT: alanine aminotransferase, TBIL: total bilirubin, ALB: albumin, PT: Prothrombin Time, PLT: platelet.

Table S3: the clinicopathological features of patients in the study after PSM

Variables	HCC patients with PVTT		
	Low ECs density (n=52)	High ECs density (n=500)	P
Age	50.31±9.867	50.50±10.722	0.924
Sex			0.339
Male	48 (92.31%)	45 (86.54%)	
Female	4 (7.69%)	7 (13.46%)	
HBeAg			1.000
Negative	40 (76.92%)	40 (76.92%)	
Positive	12 (23.08%)	12 (23.08%)	
HBV DNA			0.395
$\leq 10^4$	34 (65.38%)	38 (73.08%)	
$>10^4$	18 (34.62%)	14 (26.92%)	
AFP (ng/dL)			0.909
≤20	19 (36.54%)	18 (34.62%)	
200-400	15 (28.85%)	16 (30.77%)	
⩾400	18 (34.62%)	18 (34.62%)	
ALT (U/L)			0.554
≤44	22 (42.31%)	25 (48.08%)	
>44	30 (57.69%)	27 (51.92%)	
TBIL(umol/L):< 20.52 vs ≥ 20.52	16.23±6.697	17.72±6.762	0.172
ALB (g/L)			0.674
≤35	50 (96.15%)	48 (92.31%)	
>35	2 (3.85%)	4 (7.69%)	
PT			0.478
>13	10 (19.23%)	13 (25.00%)	
≤13	42 (80.77%)	39 (75.00%)	
PLT			0.089
>100	40 (76.92%)	32 (61.54%)	
≤100	12 (23.08%)	20 (38.46%)	
Tumor diameter (cm)			0.767
≤5	6 (11.54%)	7 (13.46%)	
>5	46 (88.46%)	45 (86.54%)	
No of tumors			0.610
Solitary	49 (94.23%)	51 (98.08%)	
Multiple	3 (5.77%)	1 (1.92%)	
Tumor capsule			0.974
Complete	33 (63.46%)	32 (61.54%)	
Incomplete	6 (11.54%)	6 (11.54%)	

Variables	HCC patients with PVTT		
	Low ECs density (n=52)	High ECs density (n=500)	P
Absent	13 (25.00%)	14 (26.92%)	
Cirrhosis			0.671
No	17 (32.69%)	15 (28.85%)	
Yes	35 (67.31%)	37 (71.15%)	
BCLC			0.710
0	1 (1.92%)	2 (3.85%)	
A	48 (92.31%)	49 (94.23%)	
B	3 (5.77%)	1 (1.92%)	
Child Pugh			
A	52 (100.00%)	52 (100.00%)	
B	0 (0.00%)	0 (0.00%)	
C	0 (0.00%)	0 (0.00%)	

Table S4: Univariate analyses of factors associated with survival and recurrence for HCC patients with PVTT before PSM

Features	OS, P value	DFS, P value
Age: <45 vs ≥45 years	0.400	0.110
Gender: female vs male	0.533	0.854
HBsAg	0.037	0.008
HBV DNA: $\leq 10^4$ vs $>10^4$	<0.001	<0.001
AFP (ng/dL): ≤ 20 vs 200 - 400 vs > 200	0.011	<0.001
ALT (U/L): ≤ 44 vs > 44	0.766	0.698
TBIL (umol/L): < 20.52 vs ≥ 20.52	0.677	0.917
ALB (g/L): ≤ 35 vs > 35	0.840	0.547
PT: ≤ 13 vs > 13	0.801	0.513
PLT: ≤ 100 vs > 100	0.748	0.681
Tumor diameter: >5 cm vs ≤5 cm	0.005	0.017
No of tumors: Solitary vs Multiple	0.846	0.830
Tumor encapsulation: complete vs none	0.002	0.002
Cirrhosis	0.020	0.002
BCLC: 0 vs A vs B	0.465	0.432
Child Pugh: A vs B	0.030	0.044
Intratumoral ECs density: high vs low	0.023	0.010

Table S5: Multivariate analyses of factors associated with overall survival and recurrence for HCC patients with PVTT before PSM

Features	OS				DFS			
		Multivariate				Multivariate		
	P	Hazard Ratio	95% CI	P	P	Hazard Ratio	95% CI	P
HBsAg	0.037				0.008			NS
HBV DNA: $\leq 10^4$ vs $>10^4$	<0.001	1.281	1.061-1.545	0.010	<0.001	1.618	1.343-1.949	<0.001
AFP (ng/dL): ≤ 20 vs 200 - 400 vs > 200	0.011	1.469	1.074-2.010	0.016	<0.001	1,537	1.187-1.990	0.001
Tumor diameter: >5 cm vs ≤5 cm	0.005	1.360	1.062-1.741	0.015	0.017			NS
Tumor encapsulation: complete vs none	0.002	0.650	0.481-0.869	0.004	0.002	0.653	0.529-0.806	<0.001
Cirrhosis	0.020			NS	0.002	1.330	1.806-1.628	0.006
Child Pugh: A vs B	0.030	1.901	1.112-3.247	0.019	0.044	1.742	1.038-2.923	0.036
Intratumoral ECs density: high vs low	0.023			NA	0.010	1.379	1.004-1.894	0.047

Table S6 Univariate analyses of factors associated with survival and recurrence for HCC patients with PVTT after PSM

Features	OS, P value	DFS, P value
Age: <45 vs ≥45 years	0.440	0.258
Gender: female vs male	0.453	0.371
HBsag	0.258	0.390
HBV DNA: $\leq 10^4$ vs $>10^4$	0.218	0.057
AFP (ng/dL): ≤ 20 vs 200 - 400 vs > 200	0.011	0.002
ALT (U/L): ≤ 44 vs > 44	0.557	0.591
TBIL (umol/L): ≤ 20.52 vs > 44	0.127	0.310
ALB (g/L): ≤ 35 vs > 35	0.641	0.637
PT: ≤ 13 vs > 13	0.599	0.276
PLT: ≤ 100 vs > 100	0.931	0.799
Tumor diameter: >5 cm vs ≤5 cm	0.632	0.389
No of tumors: Solitary vs Multiple	0.299	0.651
Tumor encapsulation: complete vs none	0.016	0.027
Cirrhosis	0.365	0.759
BCLC: 0 vs A vs B	<0.001	0.012
Intratumoral ECs density: high vs low	0.017	0.017

Table S7: Multivariate analyses of factors associated with overall survival and recurrence for HCC patients with PVTT after PSM

	OS				DFS			
		Multivariate				Multivariate		
Features	P	Hazard Ratio	95% CI	P	P	Hazard Ratio	95% CI	P
AFP (ng/dL): ≤ 20 vs 200 - 400 vs > 200	0.011	1.830	1.073-3.120	0.026	0.002	2.590	1.501-4.470	<0.001
Tumor encapsulation: complete vs none	0.016	0.534	0.320-0.890	0.016	0.027			NS
BCLC: 0 vs A vs B	<0.001	0.060	0.005-0.186	<0.001	0.012	0.145	0.028-0.765	0.009
Intratumoral ECs density: high vs low	0.017	1.604	1.047-2.458	0.030	0.017	1.919	1.240-2.969	0.003

Table S8: the relational between ECs density and the PVTT in HCC patients

Variables	Low ECs density	High ECs density	P value
PVTT			<0.001
Positive	52 (9.42%)	500 (90.58%)	
Negative	226 (24.59%)	693 (75.41%)	

Table S9: The Cumulative DFS and OS rate of HCC patients with PVTT before PSM and after PSM in the high ECs density and low ECs density groups

Variables	Before PSM		After PSM	
	**High ECs density (n=500)**	**Low ECs density (n=52)**	**High ECs density (n=52)**	**Low ECs density (n=52)**
Median DFS **Time(95%CI), months**	4.50 (4.07 - 4.90)	9.28 (7.97 - 10.20)	4.35 (2.87 - 6.83)	9.18 (6.65 - 12.62)
DFS, %				
**1-year**	24.80	38.89	25.00	38.89
**3-year**	9.86	15.95	5.77	15.95
**5-year**	6.63	10.64	5.77	10.64
P value	0.009		0.016	
Median OS **time (95%CI), months**	9.28 (7.97 - 10.20)	13.91 (10.57 - 21.46)	9.78 (6.13 - 13.20)	13.91 (10.57 - 21.46)
OS, %				
**1-year**	39.60	61.18	40.38	61.18
**3-year**	16.55	21.71	12.69	21.71
**5-year**	11.39	17.10	9.78	17.10
P value	0.022		0.016	

Table S10: Relationships between intratumoral AXL expression in EC and clinicopathological features

Variables	Intratumoral AXL expression in TEC (n=261)			Peritumoral AXL expression in NEC (n=261)		
	High (n = 108)	Low (n = 153)	*P* value	High (n = 134)	Low (n = 127)	P value
	No. of patients	No. of patients		No. of patients	No. of patients	
Age, years†	51.12 ± 10.51	54.07 ± 11.20	0.033	53.19 ± 11.82	52.48 ± 10.01	0.601
Gender			0.183			0.022
Male	85	131		118	98	
Female	23	22		16	29	
HBSAG			0.100			0.049
Positive	97	124		107	114	
Negative	10	26		24	12	
α-fetoprotein			0.900			0.137
≤200ng/dL	56	81		64	73	
>200 ng/dL	52	72		70	54	
Liver cirrhosis			1.000			
Yes	28	39		24	43	
No	80	114		110	84	
Tumor size, cm†	6.47 ± 4.65	5.04 ± 3.2	0.006	5.93 ±3.92	5.32 ± 3.92	0.208
Tumor encapsulation			0.379			0.620
Complete	50	81		65	66	
None	56	72		68	60	
Microvascular invasion			0.026			0.205
Yes	51	52		58	45	
No	56	101		75	82	
Tumor differentiation			0.009			0.589
I-II	67	118		97	88	
III-IV	41	35		37	39	
TNM stage			0.013			0.038
I	51	93		65	79	
II	43	37		46	34	
IIIA	12	23		23	12	

†Student's t-test.

‡0 percent of all cells have an expected count of less than 5; Fisher's exact test.

§Equal variances not assumed.

Table S11: Univariate analyses of factors associated with survival and recurrence

Features	OS, P value	DFS, P value
Age: <55 vs ≥55 years	0.808	0.947
Gender: female vs male	0.439	0.432
HBsag	0.099	0.352
Liver cirrhosis: yes vs no	0.245	0.221
α-fetoprotein: >200ng/dL vs ≤200ng/dL	0.019	0.009
Tumor differentiation: III-IV vs I-II	0.012	0.087
Tumor size: >5 cm vs ≤5 cm	<0.001	0.001
Tumor encapsulation: complete vs none	0.142	0.368
Microvascular invasion: yes vs no	<0.001	0.001
TNM stage: IIIA vs II vs I	<0.001	0.001
Intratumoral AXL high vs low	0.009	0.013
Peritumoral AXL high vs low	0.254	0.013

Table S12: Multivariate analyses of factors associated with overall survival and recurrence

	OS				DFS			
		Multivariate				Multivariate		
Features	P	Hazard Ratio	95% CI	P	P	Hazard Ratio	95% CI	P
α-fetoprotein: >200ng/dLvs≤200ng/dL	0.019			NS	0.009			NS
Tumor differentiation: III-IV vs I-II	0.012	4.277	0.383-0.942	0.040	0.087			NA
Tumor size: >5 cmvs≤5 cm	< 0.001	7.413	0.261-0.738	0.002	< 0.001	3.840	0.431-1.000	0.011
Microvascular invasion: yes vs no	< 0.001			NS	< 0.001			NS
TNM stage: IIIA vs II vs I	< 0.001	16.515	0.138-0.699	< 0.001	< 0.001	18.144	0-0.935	0.004
Intratumoral AXL: high vs low	0.009	4.267	0.405-0.962	0.033	0.013	4.658	0.463-0.964	0.032
Peritumoral AXL: high vs low	0.254			NA	0.041			NS

Supplementary Materials and Methods

Patients for clinical analysis and follow-up

The clinicopathological features of HCC patients who received R0 curative resections at the Eastern Hepatobiliary Surgery Hospital from 2003 and 2013 were retrospectively reviewed. The entry criteria included: (1) no preoperative or postoperative treatment, (2) no residual tumors based on inspection and histological examination of the margins after resection. (3) PVTT confirmed by preoperative imaging and observations during the operation and by pathological diagnosis, And (4) no severe preoperative dysfunction. Patients who were lost to follow-up within two months after hospital discharge or died due to complications were excluded from our study. The classification of the EC density (marked by CD34) was evaluated by pathologists based on the amount of positive tumor vasculature and the intensity of the staining.

The patients were followed-up every 2 to 3 months until dropout or death occurred. If recurrence was confirmed, patients received appropriate treatments, such as TACE, liver resection and so on, depending on the condition of the patient. The diagnosis of recurrence was evaluated according to ultrasound scanning, magnetic resonance imaging, computed tomography and raised serum a-fetoprotein (AFP) levels. Overall survival (OS) was defined as the duration from resection to the time of last follow-up or death. Recurrence-free survival (RFS) was defined as the duration from resection to the time of recurrence.

Patients for tissue microarray and follow-up

A total of 305 specimens were used. All patients received curative resection between 2003 and 2013 at the Eastern Hepatobiliary Surgery Hospital and did not receive any anti-cancer therapy before surgery. The curative resection criteria were defined as macroscopically complete removal of the tumor as described previously [(1)]. Tumor stage was assessed according to the 7th edition of the AJCC/UICC TNM classification

system[2]. Tumor differentiation was determined by the Edmondson grading system. Follow-up procedures and postoperative treatment modalities were carried out according to a uniform guideline as described in a previous study [1]. The median observation time was 32.27 months (range, 1.0–69.8). This study was approved by the ethics committee of the Eastern Hepatobiliary Surgery Hospital (Shanghai, China) and all patients provided their written informed consent to participate in this study.

AXL transfection

The lentivirus with the induced AXL expression vector, reduced AXL expression vector and negative control vector were obtained from Genechem (GeneChem, Shanghai, China) and generated in HEK293 cells. These vectors were transfected into 293-T cells using Lipofectamine 2000 (Invitrogen) and HUVECs were transfected with the virus following the manufacturer's instructions. Western blotting was used to measure AXL expression for validation. The most effective oligonucleotides for AXL (5′-GACGAAAUCCUCUAUGUCAdTdT-3′, sense) were used for the study.

siRNA and transfection

Cells were transfected with either a nonspecific control or AXL small interfering RNA (siRNA) (Sigma-Aldrich, St. Louis, MO, USA), CCL14 siRNA (Santa Cruz, Santa Cruz, CA) and Dickkopf-1 (DKK-1) siRNA (Genepharma, Shanghai, China) according to the manufacturer's instructions. The sequences of siRNA are listed in **Table S1.**

EC tube formatting assays

As described in our previous study[3], ECs (1.5×10^4 /well) were added to Matrigel-coated 96-well plates and incubated at 37°C for 6 h with CMs. Representative

photographs were taken by inverted microscopy. Tube formation was assessed by measuring the length of the tube at × 200 magnification. Relevant effects of CM were normalized according to total cellular protein.

Proliferation assay and cell migration

Cell proliferation was assayed using CCK-8 solution (Dojindo, Kumamoto, Japan) and quantitative cell migration assays were performed using a chamber (Corning, Tewksbury. MA) with an 8.0-μm polycarbonate filter inserted into a 24-well plate, as previously described (4). Relevant effects of CM were normalized according to total cellular protein.

Single cell RNA-seq workflow

Tumor or nonmalignant tissues were dissociated to a single cell solution as previously described (5). Briefly, tissues were cut into 1-mm^2 pieces and digested in dissociation buffer (RPMI-1640 medium supplied with 2% FBS, 0.05 mg/ml collagenase I, 0.05 mg/ml collagenase IV, 0.025 mg/ml hyaluronidase and 0.01 mg/ml DNase I) at 37 °C for 30 min. After stopping the digestion with excess RPMI-1640 medium and lysing RBCs, the cell strainer-filtered single cell solution was maintained on ice until loading to a BD Rhapsody cartridge for single cell transcriptome isolation.

Raw reads for a single cell transcriptome were processed through a whole transcriptome assay analysis pipeline, which included mapping reads to the Genome Reference Consortium Human Build 38 patch release 7 (GRCh38.p7) using Star (version 2.5.2b), annotating molecules with the recursive substitution error correction (RSEC) and distribution-based error correction (DBEC) algorithms, and annotating the results in accordance with GENCODE Release 25 (GRCh38.p7). The pipeline generated gene expression matrices corrected by RSEC and DBEC algorithms, which were used for subsequent clustering analysis. Cells that were determined as multiples

were excluded from subsequent procedures.

Raw gene expression matrices were read into R (version 3.5.1) and converted to a Seurat object using the Seurat R package (version 2.3.4). Cells with more than 60% UMIs derived from the mitochondrial genome were removed. As a result, 3605 cells remained after the filtering step. The gene expression matrix was then normalized to the total cellular UMI count. Highly variable genes were selected as having log normalized average gene expressions between 0.05 and 3 and dispersion levels above 0.5. After scaling the data with respect to UMI counts, PCA was performed based on the highly variable genes identified in the previous step to reduce dimensionality. In addition, the first 15 principle components were chosen based on a PC heat map, a Jackstraw plot, and a PC elbow plot to further reduce dimensionality using the tSNE algorithm. Eleven clusters were identified with the default setting using the RunTSNE function. Each cluster was then annotated with the canonical cell markers.

To further analyze endothelial cells, cells from Cluster 3 and Cluster 8 with a positive CD31 expression were extracted. Then, the AXL expression levels for different clusters were plotted.

Statistical analysis

SPSS 22.0 for Windows (SPSS Inc., Chicago, IL, USA) was used for analysis in this study. Continuous data were compared using Mann-Whitney U tests; normally distributed data were compared using Student's t tests. Categorical variables were compared using Chi-squared tests or Fisher's exact tests. OS or DFS were analyzed using the Kaplan-Meier method and compared with the log-rank test. Univariate and multivariate analyses were assessed using a Cox proportional hazards stepwise model. Because there was a heterogeneity between the high CD34 group and low CD34 group in the study population, propensity score matching (PSM) was performed. PSM was performed on an alpha-fetoprotein (AFP) level and PSM. $P < 0.05$ was considered statistically significant.

Supplementary Results

Patient characteristics

A total of 3388 patients were diagnosed as having HCC with PVTT at the Eastern Hepatobiliary Surgery Hospital from 2003 to 2013. A total of 2836 patients were excluded because they did not receive a resection (n = 2236), had incomplete follow-up data (n = 286), had preoperative treatments (n = 287), or died due to postoperative complications (n = 15). Finally, a total of 552 patients were enrolled in this study. Before PSM, there were 500 patients in the high CD34 group and 52 patients in the low CD34 group. PSM matched 52 patients in each. The baseline characteristics of the patients in the two groups (**Table S2**) showed the high CD34 group had higher serum AFP levels ($P < 0.001$) than the low CD34 group.

The clinicopathological features of patients in the study after PSM are shown in **Table S3.**

Risk factors for poor RFS and OS

Univariate and multivariate analyses before PSM demonstrated that HBV-DNA ($P = 0.010$), serum AFP levels ($P = 0.016$), tumor diameter ($P = 0.015$), tumor encapsulation ($P = 0.004$) and Child Pugh B ($P = 0.019$) were significant but were not independent risk factors of poor OS. However, HBV-DNA ($P < 0.001$), serum AFP level ($P = 0.001$), tumor encapsulation ($P < 0.001$), cirrhosis ($P = 0.006$), Child Pugh B ($P = 0.036$) and high intratumoral EC density ($P = 0.047$) were independent risk factors for poor DFS (**Table S4 and S5**). Univariate and multivariate analyses after PSM demonstrated that serum AFP levels ($P = 0.026$), tumor encapsulation ($P = 0.016$), Child Pugh B ($P < 0.001$) and high intratumoral EC density ($P = 0.030$) were independent risk factors of poor OS, whereas serum AFP levels ($P < 0.001$), Child Pugh B ($P = 0.009$) and high intratumoral EC density ($P = 0.003$) were independent risk factors for poor DFS (**Table S8 and S9**).

Survival analysis

Before PSM, the median OS times (MOST 95% CI) of the two groups of patients after resection were 9.28 (7.97–10.20) months for the high intratumoral EC density group and 13.91 (10.57–21.46) months for the low intratumoral EC density group. OS was significantly better for patients in the low intratumoral EC density group than the high intratumoral EC density group (1 year, 61.18% vs 39.60%; 3 years, 21.71% vs 16.55%; and 5 years, 17.10% vs 11.39%; P = 0.022). The median DFS times (MDFST 95% CI) of the two groups of patients after resection were 4.50 (4.07–4.90) months for the high intratumoral EC density group and 9.28 (7.97–10.20) months for the low intratumoral EC density group. DFS was significantly better for patients in the low intratumoral EC density group than the high intratumoral EC density group (1 year, 38.89% vs 24.80%; 3 years, 15.95% vs 9.86%; and 5 years, 10.64% vs 6.63%; P = 0.009, **Fig. 1A and Table S13**).

After PSM, the MOST (95% CI) of the two groups of patients after resection was 9.78 (6.13-13.20) months for the high intratumoral EC density group and 13.91 (10.57-21.46) months for the low intratumoral EC density group. OS was significantly better for patients in the low intratumoral EC density group than the high intratumoral EC density group (1 year, 61.18% vs 40.38%; 3 years, 21.71% vs 12.69%; and 5 years, 17.10% vs 9.78%; P = 0.016). The MDFST (95% CI) of the two groups of patients after resection were 4.35 (2.87-6.83) months for the high intratumoral EC density group and 9.18 (6.65-12.62) months for the low intratumoral EC density group. DFS was significantly better for patients in the low intratumoral EC density group than the high intratumoral EC density group (1 year, 38.89% vs 25.00%; 3 years, 15.95% vs 5.77%; and 5 years, 10.64% vs 5.77%; P = 0.016, **Fig. 1B and Table S13**).

References:

1. Sun HC, Zhang W, Qin LX, Zhang BH, Ye QH, Wang L, Ren N, et al. Positive serum hepatitis B e antigen is associated with higher risk of early recurrence and poorer survival in patients after curative resection of hepatitis B-related hepatocellular carcinoma. J Hepatol 2007;47:684-690.
2. Wittekind C. [2010 TNM system: on the 7th edition of TNM classification of malignant tumors].

Pathologe 2010;31:331-332.
3. Ao JY, Chai ZT, Zhang YY, Zhu XD, Kong LQ, Zhang N, Ye BG, et al. Robo1 promotes angiogenesis in hepatocellular carcinoma through the Rho family of guanosine triphosphatases' signaling pathway. Tumour Biol 2015;36:8413-8424.
4. Ye BG, Sun HC, Zhu XD, Chai ZT, Zhang YY, Ao JY, Cai H, et al. Reduced expression of CD109 in tumor-associated endothelial cells promotes tumor progression by paracrine interleukin-8 in hepatocellular carcinoma. Oncotarget 2016;7:29333-29345.
5. Song Y, Gan Y, Wang Q, Meng Z, Li G, Shen Y, Wu Y, et al. Enriching the Housing Environment for Mice Enhances Their NK Cell Antitumor Immunity via Sympathetic Nerve-Dependent Regulation of NKG2D and CCR5. Cancer Res 2017;77:1611-1622.

Fig.S1

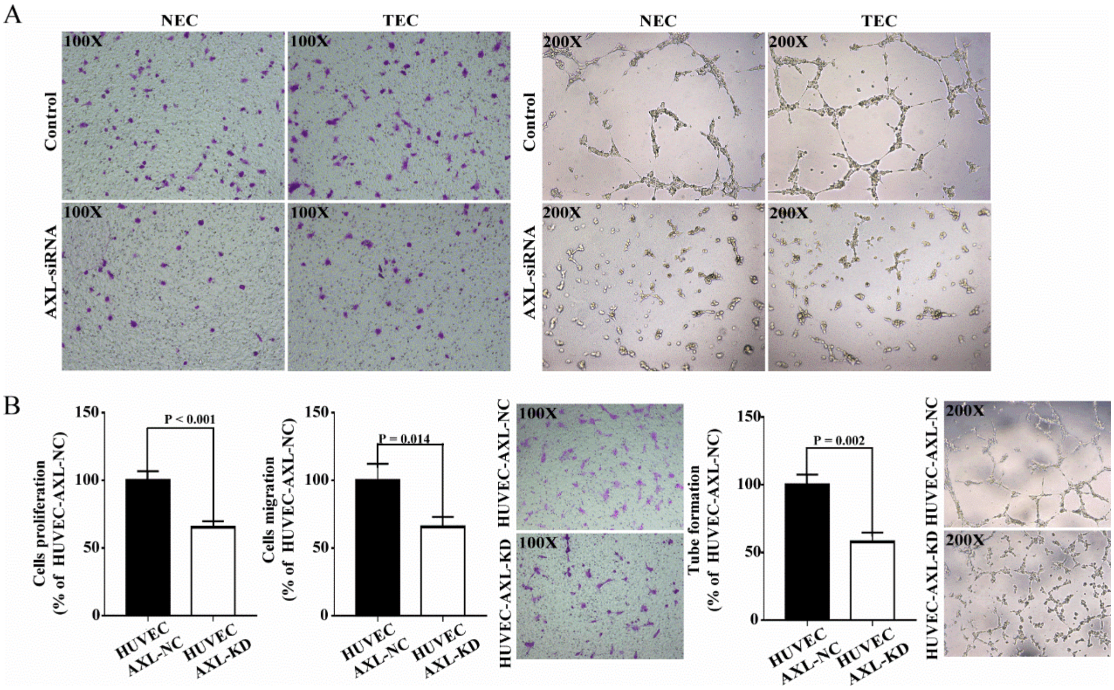

A
NEC
TEC
NEC
TEC
100X
200X
Control
AXL-siRNA
B
Cells proliferation (% of HUVEC-AXL-NC)
P < 0.001
HUVEC AXL-NC
HUVEC AXL-KD
Cells migration (% of HUVEC-AXL-NC)
P = 0.014
HUVEC-AXL-NC
HUVEC-AXL-KD
Tube formation (% of HUVEC-AXL-NC)
P = 0.002
150
100
50
0

Fig.S2

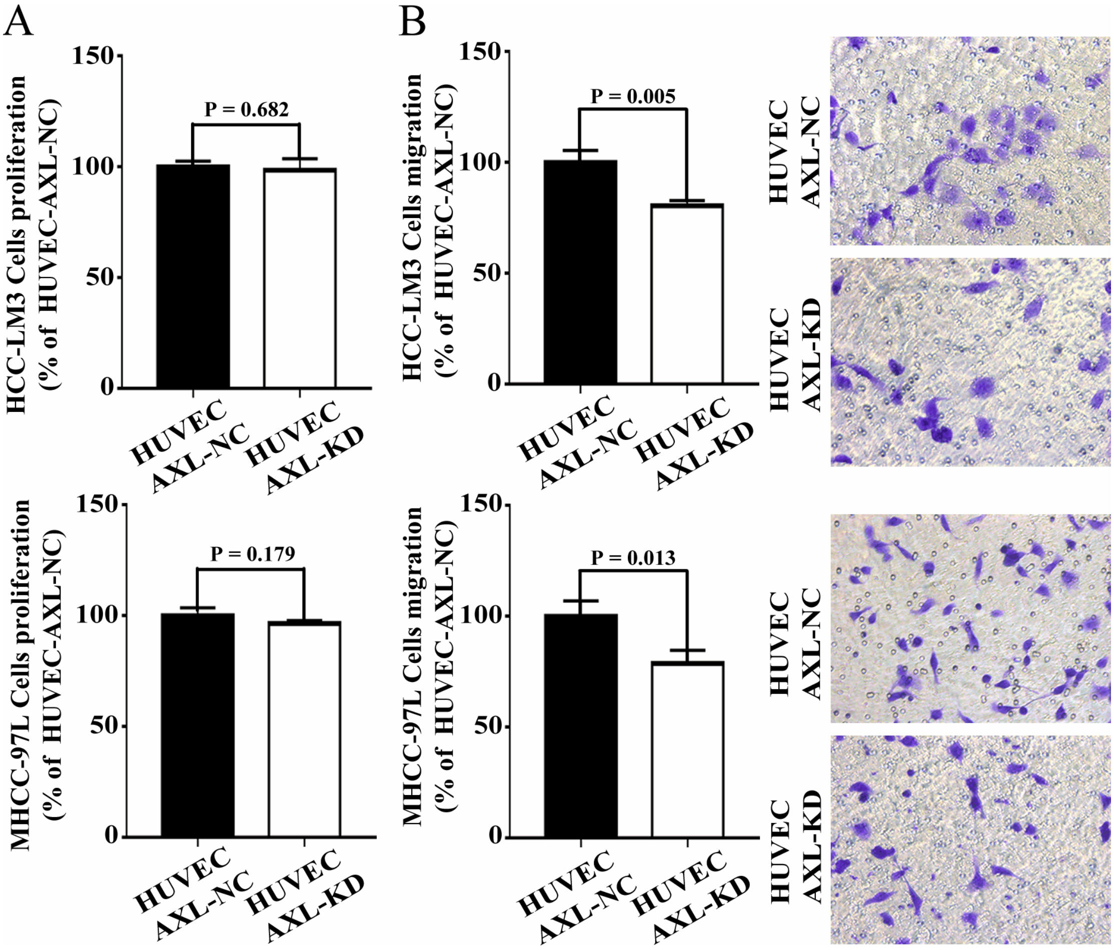
A
B
HCC-LM3 Cells proliferation (% of HUVEC-AXL-NC)
P = 0.682
HUVEC AXL-NC
HUVEC AXL-KD
HCC-LM3 Cells migration (% of HUVEC-AXL-NC)
P = 0.005
MHCC-97L Cells proliferation (% of HUVEC-AXL-NC)
P = 0.179
MHCC-97L Cells migration (% of HUVEC-AXL-NC)
P = 0.013
0
50
100
150

Fig.S3

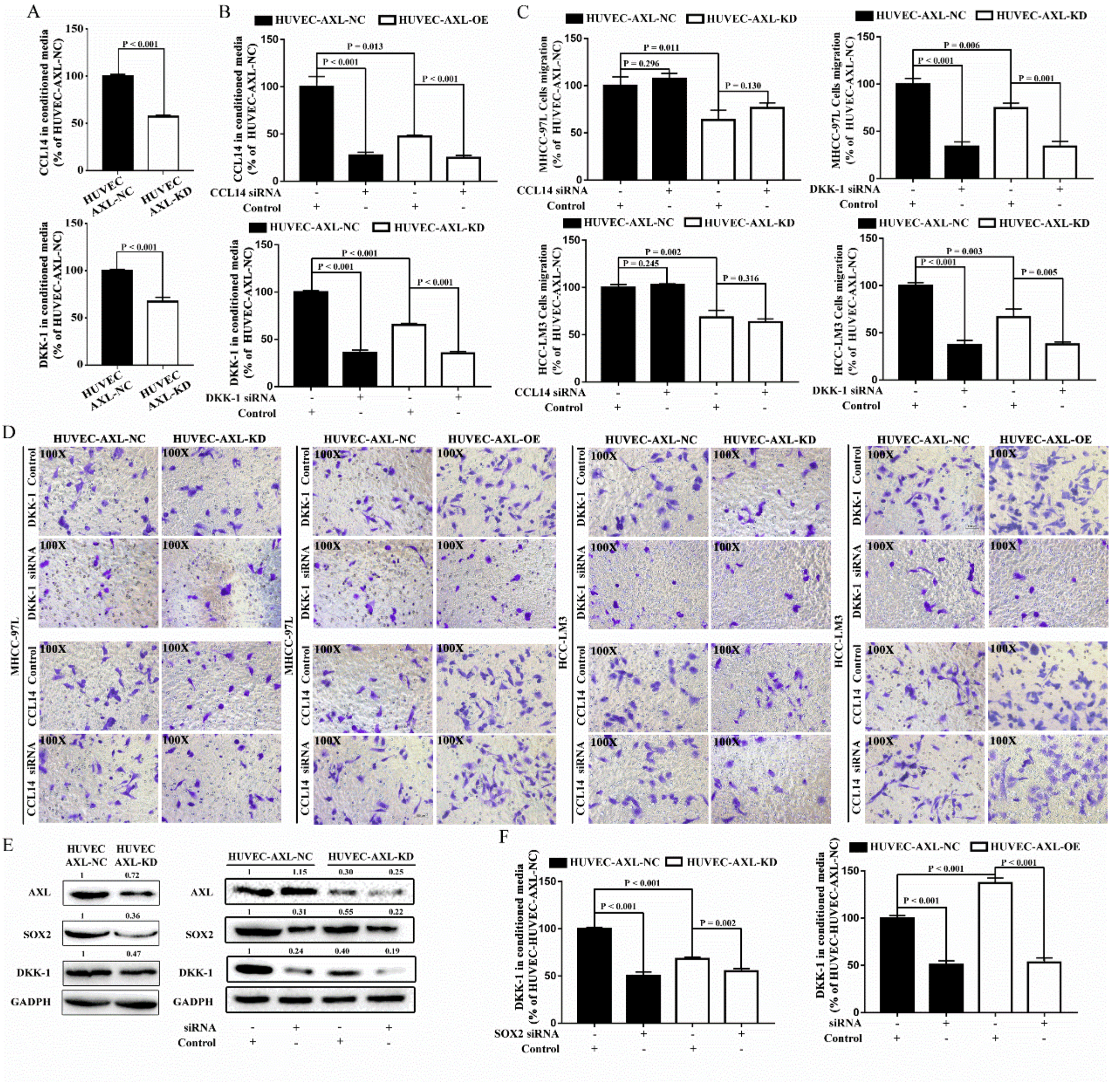
A
B
C
D
E
F
HUVEC-AXL-NC
HUVEC-AXL-KD
HUVEC-AXL-OE
CCL14 in conditioned media (% of HUVEC-AXL-NC)
DKK-1 in conditioned media (% of HUVEC-AXL-NC)
MHCC-97L Cells migration (% of HUVEC-AXL-NC)
HCC-LM3 Cells migration (% of HUVEC-AXL-NC)
DKK-1 in conditioned media (% of HUVEC-HUVEC-AXL-NC)
P < 0.001
P = 0.013
P = 0.011
P = 0.296
P = 0.130
P = 0.006
P = 0.001
P = 0.002
P = 0.245
P = 0.316
P = 0.003
P = 0.005
CCL14 siRNA
DKK-1 siRNA
SOX2 siRNA
siRNA
Control
MHCC-97L
HCC-LM3
100X
AXL
SOX2
DKK-1
GADPH

Fig.S4

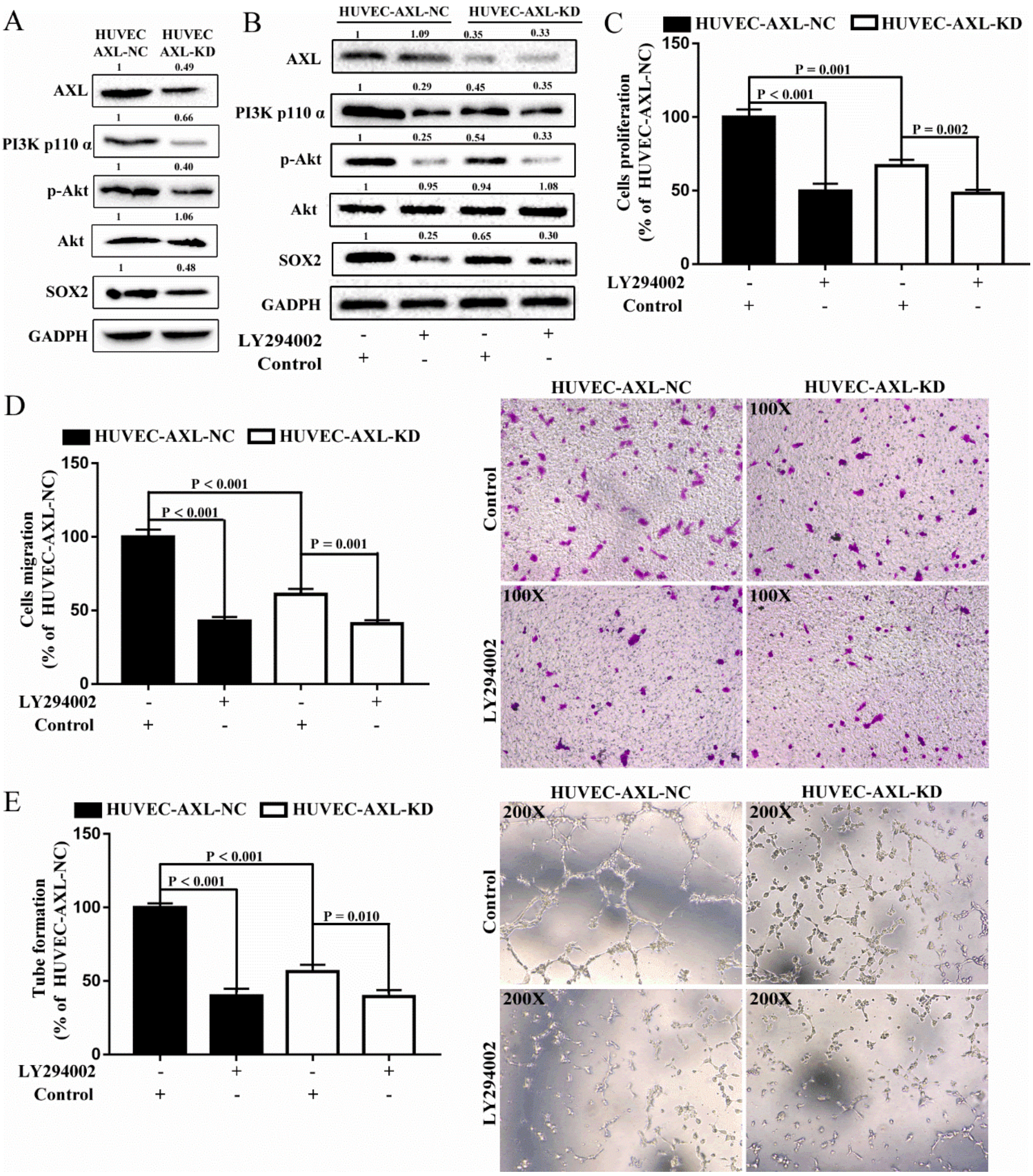
A
HUVEC AXL-NC
HUVEC AXL-KD
AXL
PI3K p110 α
p-Akt
Akt
SOX2
GADPH
B
HUVEC-AXL-NC
HUVEC-AXL-KD
LY294002
Control
C
Cells proliferation (% of HUVEC-AXL-NC)
P = 0.001
P < 0.001
P = 0.002
D
Cells migration (% of HUVEC-AXL-NC)
P < 0.001
P < 0.001
P = 0.001
100X
E
Tube formation (% of HUVEC-AXL-NC)
P < 0.001
P < 0.001
P = 0.010
200X

Fig.S5

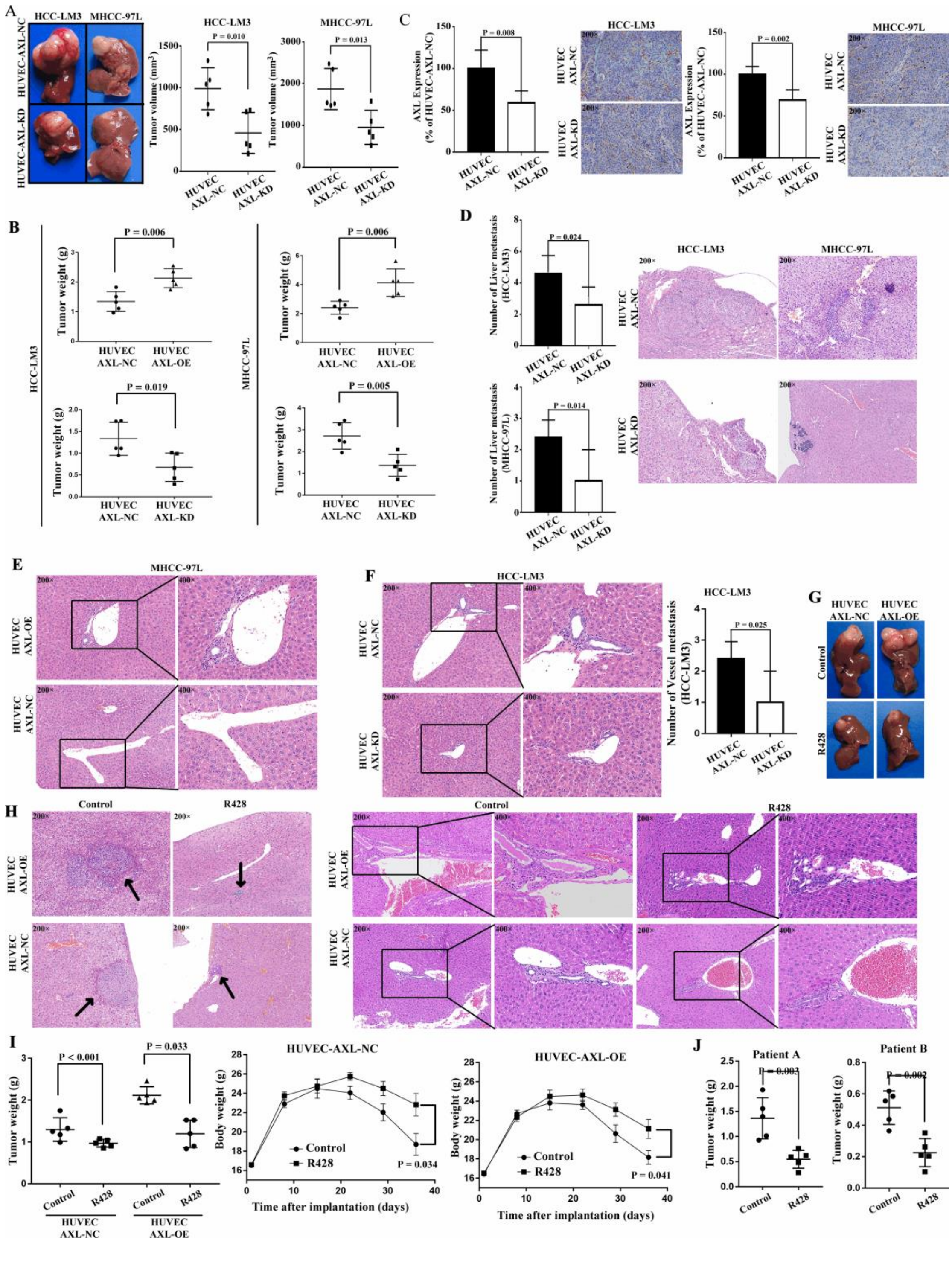

A
HCC-LM3
MHCC-97L
HUVEC-AXL-NC
HUVEC-AXL-KD
Tumor volume (mm3)
P = 0.010
P = 0.013
HUVEC AXL-NC
HUVEC AXL-KD
C
AXL Expression (% of HUVEC-AXL-NC)
P = 0.008
P = 0.002
200×
B
HCC-LM3
MHCC-97L
Tumor weight (g)
P = 0.006
P = 0.019
P = 0.005
HUVEC AXL-OE
D
Number of Liver metastasis (HCC-LM3)
Number of Liver metastasis (MHCC-97L)
P = 0.024
P = 0.014
E
F
400×
Number of Vessel metastasis (HCC-LM3)
P = 0.025
G
Control
R428
H
I
P < 0.001
P = 0.033
HUVEC-AXL-NC
HUVEC-AXL-OE
Body weight (g)
Time after implantation (days)
P = 0.034
P = 0.041
J
Patient A
Patient B
P = 0.003
P = 0.002
